# A ‘frost formation’-inspired near-infrared-responsive nitric oxide-releasing hydrogel for enhancing fat graft survival

**DOI:** 10.1093/rb/rbag086

**Published:** 2026-05-05

**Authors:** Yanglong Zhu, Yatian Wei, Zixun Lan, Yuanzheng Zhu, Hengyu Wu, Tingting Zhong, Xiang Shen, Ganghua Yang, Yangyan Yi, Xiaolei Wang

**Affiliations:** Department of Plastic Surgery, The Second Affiliated Hospital, Jiangxi Medical College, Nanchang University, Nanchang, Jiangxi 330006, China; The National Engineering Research Center for Bioengineering Drugs and the Technologies, Institute of Translational Medicine, Nanchang University, Jiangxi 330088, China; Jiangxi Province Key Laboratory of Precision Cell Therapy, Jiangxi Medical College, Nanchang, Jiangxi 330006, China; Department of Thoracic Surgery, The Second Affiliated Hospital, Jiangxi Medical College, Nanchang University, Nanchang, Jiangxi 330006, China; The National Engineering Research Center for Bioengineering Drugs and the Technologies, Institute of Translational Medicine, Nanchang University, Jiangxi 330088, China; Department of Plastic Surgery, The Second Affiliated Hospital, Jiangxi Medical College, Nanchang University, Nanchang, Jiangxi 330006, China; Jiangxi Province Key Laboratory of Precision Cell Therapy, Jiangxi Medical College, Nanchang, Jiangxi 330006, China; Department of Plastic Surgery, The Second Affiliated Hospital, Jiangxi Medical College, Nanchang University, Nanchang, Jiangxi 330006, China; Jiangxi Province Key Laboratory of Precision Cell Therapy, Jiangxi Medical College, Nanchang, Jiangxi 330006, China; School of Chemistry and Chemical Engineering, Nanchang University, Nanchang, Jiangxi 330088, China; The National Engineering Research Center for Bioengineering Drugs and the Technologies, Institute of Translational Medicine, Nanchang University, Jiangxi 330088, China; Department of Plastic Surgery, The Second Affiliated Hospital, Jiangxi Medical College, Nanchang University, Nanchang, Jiangxi 330006, China; Jiangxi Province Key Laboratory of Precision Cell Therapy, Jiangxi Medical College, Nanchang, Jiangxi 330006, China; Department of Plastic Surgery, The Second Affiliated Hospital, Jiangxi Medical College, Nanchang University, Nanchang, Jiangxi 330006, China; Jiangxi Province Key Laboratory of Precision Cell Therapy, Jiangxi Medical College, Nanchang, Jiangxi 330006, China; The National Engineering Research Center for Bioengineering Drugs and the Technologies, Institute of Translational Medicine, Nanchang University, Jiangxi 330088, China; School of Chemistry and Chemical Engineering, Nanchang University, Nanchang, Jiangxi 330088, China

**Keywords:** near-infrared light response, nitric oxide, hydrogel-based delivery system, fat graft

## Abstract

Soft tissue defects represent a major challenge in plastic surgery, for which autologous fat grafting remains the standard treatment. However, in the early post-transplant period, the grafted fat tissue undergoes ischemia and hypoxia, leading to low and markedly unpredictable survival rates that consequently compromise the aesthetic outcomes. Nitric oxide (NO), an endogenous gasotransmitter, plays a vital role in mediating angiogenesis and vascular remodeling. Therefore, we developed a near-infrared-responsive hydrogel that releases NO on demand to promote adipocyte vascularization and thereby improve graft retention and enable precise volumetric filling. To ensure high filling, the platform incorporates a photosensitive gelatin methacryloyl (GelMA) hydrogel inspired by the rapid solidification of ‘frost formation’. The hydrogel can be precisely delivered to the target area and photocrosslinked *in situ* to form a three-dimensional network that supports cell growth. Notably, this photocrosslinking process does not require ultraviolet irradiation, thereby eliminating the associated risks and significantly greatly enhancing the safety of adipose remodeling and filling technologies. Experimental studies conducted both *in vitro* and *in vivo* have validated that this hydrogel platform ensures precise grafting, promotes graft integration and significantly improves the long-term survival of transplanted adipose tissue.

## Graphical abstract

**Figure:**
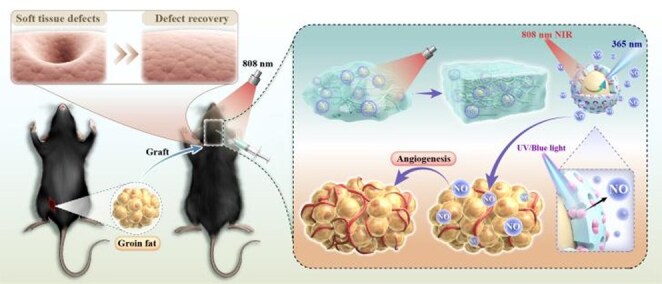

## Introduction

Soft tissue defects arise from diverse etiologies such as surgical resection, severe trauma and congenital disorders [1–3]. Such defects not only impair normal physiological function but also exert substantial negative impacts on patients’ daily activities. Autologous fat transplantation has emerged as a widely used technique for soft tissue reconstruction (applied in breast reconstruction, facial filling and scar repair) due to its abundant sources, minimal immunogenicity and minimally invasive nature [4–7]. However, the survival rate of grafted fat remains highly variable, ranging between 20% and 80% [8]. A key contributing factor is early post-transplant ischemia and hypoxia within the graft [9]. Therefore, improving graft survival and ensuring stable long-term outcomes remain the major challenges associated with the technique.

A long-term and stable blood supply is crucial to ensuring a high survival rate of transplanted tissues [10]. Nitric oxide (NO), an endogenous gas primarily generated by nitric oxide synthase (NOS), plays important roles in angiogenesis, vascular remodeling, neural signals, tissue regeneration and anti-apoptosis [11–15]. However, NO is a highly reactive gaseous molecule, featuring a half-life of only 5 s and a diffusion range confined to 40–200 μm [16]. To achieve the targeted, timely and controlled release of NO [17–20], a promising approach is to regulate the release of NO from exogenous donors in the body by precisely controlling the irradiation position, duration and power of external light. Nevertheless, the light used to activate NO donors is almost exclusively ultraviolet (UV) light [21, 22]. UV light, as a high-energy light with shallow tissue penetration, may cause skin erythema and pruritus when improperly exposed, and cumulative phototoxicity increases the risks of skin aging and carcinogenesis [23]. Therefore, the application of UV light in the field of plastic surgery is strictly limited. Thus, there is an urgent need to develop a compatible light-responsive NO delivery system activated by a milder, deeper-penetration light source to enhance angiogenesis and ultimately improve fat graft survival.

To ensure durable and stable filling outcomes, the implant must provide consistent structural support and a sustained oxygen supply to the transplanted tissue. Therefore, an ideal implant material should exhibit two essential characteristics: a precisely controllable solidification process and the ability to provide long-term, continuous nutritional support. Hydrogels are polymeric substances featuring a three-dimensional network structure, with outstanding biomimetic properties and adjustable characteristics. Thanks to their high-water content and porosity, they effectively mimic the natural extracellular matrix (ECM) structure, thereby supporting cell proliferation, adhesion and nutrient transport. Gelatin methacryloyl (GelMA) [24, 25], synthesized from gelatin and methacrylic anhydride (MA), is a biodegradable [26, 27] and photosensitive [28] hydrogel widely used in tissue engineering [29, 30]. Upon exposure to light, GelMA undergoes rapid crosslinking to form a 3D network with suitable mechanical properties for cellular growth and differentiation [31, 32]. Furthermore, GelMA hydrogel shows remarkable advantages in localized drug delivery, as it can not only shield unstable therapeutic agents from degradation but also achieve precise spatial and temporal regulation of drug release.

**Figure 1:**
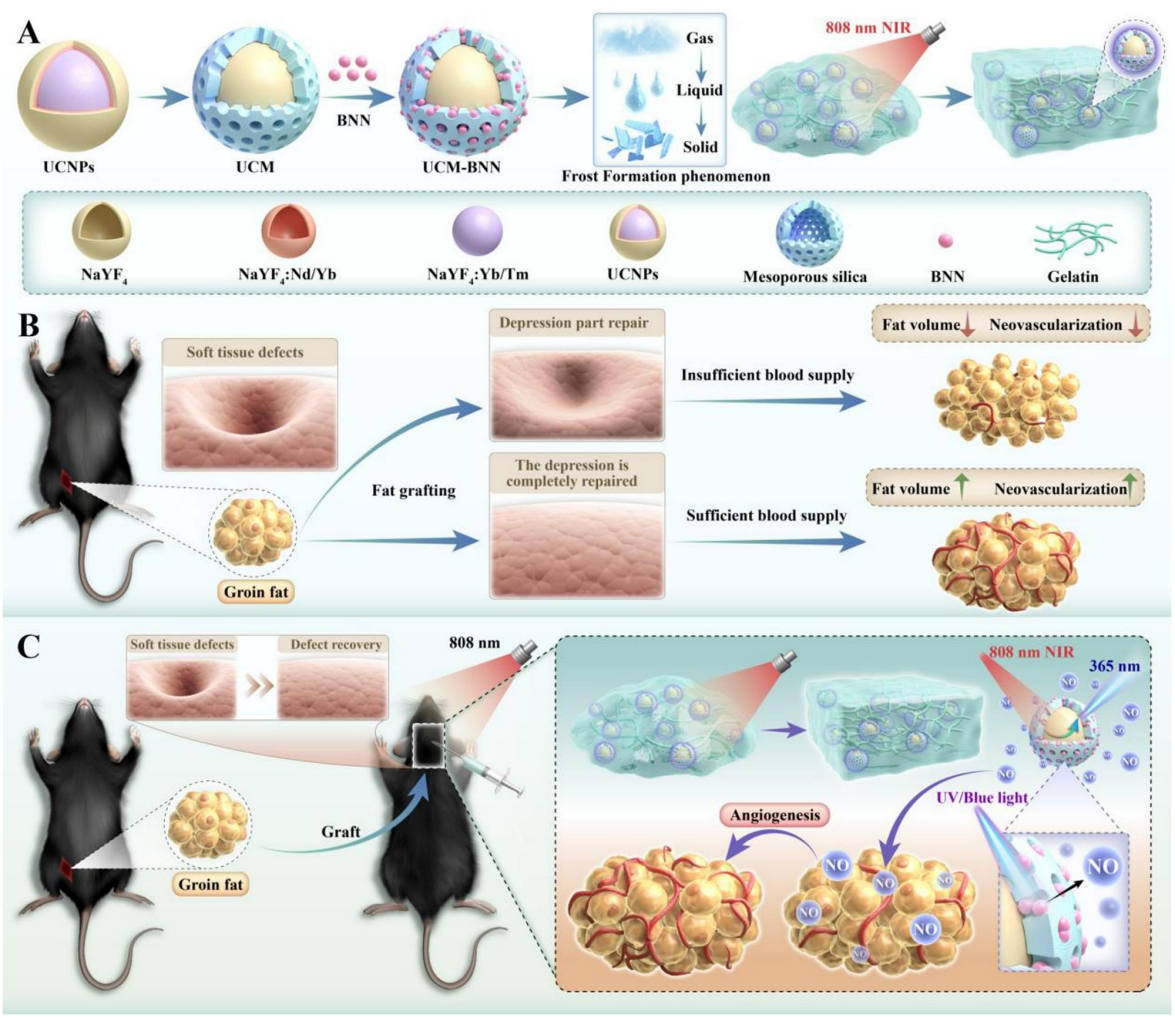
Synthesis process and working principle of Gel@UCM-BNN hydrogel. (**A**) Mesoporous silica coated doped upconversion nanoparticles (UCNPs) were further loaded with BNN to prepare UCM-BNN. After being encapsulated by GelMA hydrogel, a NO delivery system (Gel@UCM-BNN hydrogel) assembles when illuminated with 808 nm NIR light. (**B**) Outcomes of fat after repairing soft tissue defects with adipose tissue. (**C**) Inguinal fat from mice is harvested to treat soft tissue defects at the top of the head. Under 808 nm NIR light irradiation, the Gel@UCM-BNN hydrogel delivery system releases NO, which acts on the transplanted fat. This NO release promotes angiogenesis within the grafted adipose tissue, improving fat survival rate and thereby achieving the goal of repairing soft tissue defects.

To address these issues, and inspired by the quick chemical solid phenomenon of ‘frost formation’, this study synthesized a delivery platform based on GelMA (Figure 1A). ‘Frost’ is a natural phenomenon in which water vapor on the group surface desublimate into solid when encountering cold air due to temperature differences [33]. Its unique phase-transition behavior and formation mechanisms inspired the structural design and functional optimization of our delivery platform. In this study, rapid and precise filling is achieved through an instant solidification process analogous to ‘frost formation’. To promote angiogenesis of adipocytes, this platform incorporated the NO donor N, N′-di-sec-butyl-N, N′-dinitroso-1,4-phenylenediamine (BNN), which enables controllable NO release upon light stimulation. Furthermore, to overcome the limitation of conventional UV light sources (namely, their high phototoxicity and limited tissue penetration), we incorporated mesoporous-silica-modified upconversion nanoparticles (UCNPs) into the system. As illustrated in Figure 1B and C, upon excitation by safer and more tissue-penetrating near-infrared (NIR) light, the GelMA hydrogel not only rapidly crosslinks and solidifies *in vivo* but also activates the gas precursor to release NO. This process subsequently accelerates adipose tissue angiogenesis, ultimately achieving the goals of precise filling and improved post-transplantation fat survival rates. It is noteworthy that, compared to a 980 nm light source, this work utilizes 808 nm NIR light for excitation, which effectively mitigates overheating effects and further ensures the safety of the therapeutic system. To our knowledge, this represents the first application of a NIR-responsive NO-controlled release hydrogel platform in fat grafting. This study provides an important technical basis for achieving safe, long-term and precise fat transplantation therapy.

## Materials and methods

### Materials

Ytterbium(III) acetate hydrate [Yb(CH_3_CO_2_)_3_·xH_2_O, 99.95% purity], yttrium(III) acetate hydrate [Y(CH_3_CO_2_)_3_·xH_2_O, 99.9% purity], thulium(III) acetate hydrate [Tm(CH_3_CO_2_)_3_·xH_2_O, 99.9% purity] and neodymium(III) acetate hydrate [Nd(CH_3_CO_2_)_3_·xH_2_O, 99.9% purity] were acquired from Sigma-Aldrich (United States). Technical grade oleic acid (OA, 90%) and 1-octadecene (ODE, 90%) were also procured from the same supplier. Tetraethyl orthosilicate (TEOS, 99% purity) and ammonium fluoride (NH_4_F, 98% purity) were sourced from Alfa Aesar (Shanghai, China). Analytical grade hydrochloric acid (HCl, 37%) was supplied by Macklin Biochemical Co., Ltd. (Shanghai, China). DAF-FM DA and Hoechst 33342 were obtained from MedChemExpress (MCE, USA). Aladdin Biochemical Technology Co., Ltd. (Shanghai, China) supplied 2,3-diaminonaphthalene (DAN, 97%) and sodium nitrite (NaNO_2_, 99.99%). Xilong Scientific Co., Ltd. (Guangzhou, China) supplied methanol, ethanol, ammonia solution (25–30%) and cyclohexane. N, N-di-sec-butyl-p-phenylenediamine (BPA), cetyltrimethylammonium bromide (CTAB, 99%), sodium hydroxide (NaOH, 97%), gelatin, methacrylic anhydride and lithium phenylbis (2,4,6-trimethylbenzoyl) phosphinate salt were obtained from Macklin Biochemical Co., Ltd. (Shanghai, China). Without any further purification, all reagents (analytical grade) were used directly.

### Synthesis of core nanocrystals

The synthesis of NaYF_4_:25%Yb/0.5%Tm core nanocrystals was achieved through a high-temperature thermal decomposition approach as described in Ref. [16]. Initially, precise quantities of yttrium acetate, ytterbium acetate, thulium acetate and neodymium acetate were measured and individually dissolved in ultrapure water to create 0.2 mol/L stock solutions of rare-earth acetates. A combined volume of 5 mL containing 1 mmol of these rare-earth acetate solutions (with molar ratios Y:Yb:Tm = 74.5:25:0.5) was introduced into a 50 mL three-neck flask. Subsequently, 15 mL of 1-octadecene and 6 mL of oleic acid were added to the flask. The resulting cloudy mixture was heated to 150°C and maintained at this temperature for half an hour until achieving complete transparency with a light-yellow coloration, after which it was allowed to cool naturally. Upon reaching room temperature, a methanol-based solution containing 4 mmol ammonium fluoride and 2.5 mmol sodium hydroxide was rapidly introduced while maintaining vigorous agitation. Concurrently, the reaction mixture was subjected to heating. The mixture was initially heated to 48°C and maintained at this temperature for half an hour to facilitate nucleation. Subsequently, the temperature was gradually increased to 100°C to ensure complete methanol evaporation. Following this, the solution underwent rapid heating to reach 300°C, where it remained for 60 min (extended to approximately 90 min during colder seasons). Upon reaction completion, the heating apparatus was removed, allowing the mixture to naturally cool to ambient conditions. For core UCNP precipitation, an abundant quantity of pure ethanol was added, followed by centrifugation at 8000 revolutions per minute for 8 min. After removing the liquid portion, 5 mL of cyclohexane was introduced, and ultrasonic treatment was applied to achieve proper dispersion. Additional ethanol was then incorporated to reprecipitate the core UCNPs, which were subsequently redispersed in 5 mL of cyclohexane. The final colloidal suspension was stored at 4°C in refrigeration.

### Synthesis of core@shell UCNPs nanocrystals

The synthesis of NaYF_4_:25%Yb/0.5%Tm@NaYF_4_:20%Nd/10%Yb core-shell nanoparticles followed an analogous high-temperature thermal decomposition approach. Initially, a 2.5 mL solution containing 0.5 mmol of rare-earth acetates with a molar ratio of Y:Nd:Yb = 7:2:1 was prepared and introduced. This was followed by the sequential addition of 6 mL oleic acid and 15 mL 1-octadecene. The resulting cloudy mixture was heated to 150°C and maintained at this temperature for half an hour, yielding a clear, faintly yellow solution. Next, 5 mL of cyclohexane containing the pre-synthesized core UCNPs was incorporated. The system was left undisturbed until all cyclohexane had evaporated, as indicated by the absence of bubbling, before being cooled naturally to room temperature. Upon reaching ambient conditions, a methanol solution containing 2 mmol ammonium fluoride and 1.25 mmol sodium hydroxide was introduced under vigorous agitation. The reaction system was then heated to 48°C and kept at this temperature for a 30 min period. The nucleation process was allowed to proceed for 30 min. Following this initial phase, the temperature was gradually increased to 100°C to facilitate methanol evaporation. The system was then quickly heated to 300°C, where it was maintained for 60 min to enable crystalline development. Upon reaction completion, the heating source was disconnected, permitting natural cooling to ambient conditions. For nanoparticle isolation, a substantial quantity of absolute ethanol was introduced to the mixture. Centrifugation at 8000 revolutions per min for 8 min separated the solid product from the liquid phase. The collected material was then ultrasonically dispersed in 5 mL of cyclohexane. The purification procedure was repeated by adding additional ethanol to induce precipitation again. The final purified core@shell upconversion nanoparticles were stored at 4°C after being redispersed in 5 mL of cyclohexane.

### Synthesis of core@shell@shell UCNPs nanocrystals

The synthesis of NaYF_4_:25%Yb/0.5%Tm@NaYF_4_:20%Nd/10%Yb@NaYF_4_ nanocrystals with a triple-layered core-shell-shell structure was achieved using the high-temperature thermal decomposition method. Initially, a 2.5 mL solution containing 0.5 mmol of rare-earth acetates (completely composed of Y) was prepared. Subsequently, 6 mL of oleic acid and 15 mL of 1-octadecene were sequentially incorporated into the mixture. This solution was then subjected to heating at 150°C for half an hour, resulting in a transparent, light-yellow liquid. At this stage, 5 mL of core-shell upconversion nanoparticles (UCNPs) dispersed in cyclohexane were introduced into the reaction system. The cyclohexane was allowed to evaporate completely, which was confirmed by the absence of bubbles in the solution. The mixture was then left undisturbed until it cooled to ambient temperature. Once cooled, a methanol-based solution containing 2 mmol of ammonium fluoride and 1.25 mmol of sodium hydroxide was rapidly injected under vigorous stirring. Simultaneously, the temperature of the mixture was raised to 48°C and maintained for 30 min. Following nucleation, the temperature was gradually increased to 100°C to facilitate the evaporation of methanol. The temperature was rapidly increased to 300°C and held constant for 60 min. After the reaction concluded, the heating apparatus was removed. The mixture was allowed to cool to ambient conditions before introducing a surplus of dehydrated ethanol, which induced the formation of core-shell-shell UCNPs. These nanoparticles were isolated through centrifugation at 8000 rpm for 8 min, yielding a solid residue that was subsequently sonicated in 5 mL of cyclohexane. An additional ethanol treatment was performed to reprecipitate the core-shell-shell UCNPs, followed by their redispersion in 5 mL of cyclohexane. The resulting colloidal suspension (core@shell@shell) was stored at 4°C and is referred to as UCNPs for brevity.

### Synthesis of UCNP@mSiO_2_ nanoparticles

The Stöber method coated UCNPs with mesoporous silica, and the resulting material was designated UCNP@mSiO_2_ (UCM). First, 0.1 g CTAB was dissolved in 20 mL deionized water with heating and stirring. Then, 2 mL of UCNPs dispersed in cyclohexane was introduced. After vigorous stirring in a preheated 80°C water bath until near clarity, the solution was sonicated to become completely transparent. Immediately afterward, the solution was drawn into a 50 mL syringe and filtered (0.22 μm disposable membrane) into a 250 mL three-necked flask. The sequential addition of 20 mL ultrapure water and 3 mL anhydrous ethanol was followed by titration with 2 M aqueous sodium hydroxide to reach pH 10–11. The mixture was then moved to a water bath preheated to 70°C and stirred at high speed. Upon temperature stabilization, the pre-prepared TEOS ethanol solution (10% v/v) was slowly injected into the three-necked flask with a 1 mL syringe, and this condition was maintained for 0.5 h. During the dropping process, the solution color was observed to gradually change from clear and colorless to pale blue, then to dark blue and finally to blue–white. This indicated that with the addition of TEOS, the mesoporous silica layer around UCNPs grew progressively thicker. When the solution turned blue, the dropping of TEOS was stopped, and heating was terminated simultaneously. After the reaction was completed, centrifugation of the solution at 11 000 rpm for 50 min yielded UCNP@mSiO_2_, which was rinsed repeatedly with anhydrous ethanol. Finally, the template agent CTAB was removed by refluxing overnight in a 1 wt% sodium chloride methanol solution. The product was rinsed again several times with anhydrous ethanol, and after lyophilization, a white flour-like product was obtained.

### Preparation of BNN

The preparation of BNN was carried out according to the literature [10]. Specifically, 4.68 mL of BPA was mixed with 40 mL of ethanol while maintaining constant agitation. An argon atmosphere was established to prevent oxidation before slowly introducing 40 mL of 6 M sodium nitrite solution in water. The mixture was stirred for half an hour, followed by the gradual addition of 20 mL of 6 M hydrochloric acid aqueous solution. The reaction mixture was then kept in darkness with continuous stirring for 6 h, during which a dark brown precipitate formed and agglomerated. The resulting precipitate was separated by centrifugation and sequentially cleaned using a 50% ethanol solution followed by purified water. The final product was obtained as a yellow powder after undergoing vacuum freeze-drying in the absence of light.

### Loading of BNN into UCM

Based on previous experimental results, the optimal loading ratio of UCM to BNN is 1:2 [18]. For the loading of BNN (NO donor), first, 5 mg of UCM was accurately weighed into a 20 mL brown vial. Then, a 10 mg/mL anhydrous ethanol solution of BNN was prepared and added to the vial containing UCM. Subsequently, the UCM mixture was sonicated to achieve dispersion, followed by 24 h of constant stirring. Finally, the mixed solution was aspirated and centrifuged to isolate UCM-BNN, followed by washing with anhydrous ethanol and ultrapure water, respectively.

### Preparation of methacrylated gelatin hydrogel

Dissolve 0.5 g of precisely weighed lyophilized methacrylated gelatin in 9 mL of phosphate-buffered saline (PBS). After melting the mixture in a 70°C water bath and waiting for the dense foam to disappear, the solution is sterilized by filtration (0.22 μm pore size) to produce a sterile hydrogel solution. Subsequently, 1 mL of lithium phenyl(2,4,6-trimethylbenzoyl)phosphinate salt photoinitiator was introduced to prepare a 5% (w/v) methacrylated gelatin hydrogel, which was then crosslinked by UV light irradiation for 60 s.

### Synthesis of Gel@UCM-BNN hydrogel

To synthesize the nanocomposite hydrogel, a certain amount of UCM-BNN nanoparticles were added to a 5% (w/v) GelMA precursor solution. After magnetic stirring for 2 h, the Gel@UCM-BNN prepolymer was prepared. Crosslinking was performed under NIR irradiation to obtain the UCM-BNN nanocomposite hydrogel, which was denoted as Gel@UCM-BNN for subsequent experiments.

### Characterization

The structural features of the fabricated nanoparticles were examined using transmission electron microscopy (TEM, Tecnai G2 20, Thermo Fisher Scientific) alongside scanning electron microscopy (SEM, Sigma 300, ZEISS). Fourier transform infrared (FTIR) spectroscopy (Nicolet 5700, Thermo Fisher Scientific) was utilized to investigate the molecular composition and identify key functional groups present in UCM, BNN, UCM–BNN, GelMA and Gel@UCM–BNN samples. Particle size distributions for various UCNP configurations (including core, core–shell and core–shell–shell structures) as well as UCM and UCM–BNN were measured through dynamic light scattering (DLS) techniques. Luminescence properties were assessed with a fluorescence spectrometer to record upconversion emission profiles. X-ray diffraction (XRD, D8 Advance, Bruker) analysis provided insights into crystalline phase characteristics. Surface properties of UCM were characterized through Brunauer–Emmett–Teller (BET) surface area measurements, with additional pore-size distribution data obtained from nitrogen adsorption–desorption isotherm studies (Autosorb-iQ, Quantachrome). Optical absorption characteristics were evaluated using UV–Vis spectroscopy. The optical absorption characteristics of UCM, BNN and UCM-BNN composites were analyzed using a UV-2600 spectrophotometer from Shimadzu. Structural verification of the hydrogel was conducted through 1H NMR spectroscopy at 300 MHz (Bruker). Thermal imaging equipment was employed to evaluate photothermal conversion efficiency. Microscopic examination of cell morphology and staining patterns was performed with Olympus and Leica DM i8 fluorescence microscopes. For cellular viability measurements, CCK-8 assays were processed with a VICTOR Nivo 3S microplate reader (PerkinElmer), whereas NO concentrations in both cellular environments and culture media were quantified using standardized commercial test kits.

### Assessment of NO release property *in vitro*

UCM-BNN and Gel@UCM-BNN suspensions were individually prepared in phosphate-buffered saline (PBS) at pH 7.4, creating test solutions with concentrations of 0 μg/mL, 500 μg/mL and 1 mg/mL. Under controlled dark conditions, the samples were exposed to 808 nm NIR radiation with an intensity of 1.5 W/cm^2^. Continuous NIR illumination was maintained for both UCM-BNN and Gel@UCM-BNN solutions at their designated concentrations. At 10-min intervals beginning from time zero, aliquots of the supernatant were collected during irradiation and analyzed using a nitric oxide detection assay. The calibration curve for sodium nitrite (NaNO_2_) was established following the precise protocols outlined in the NO detection kit’s manual. Following incubation of the supernatant with the kit’s working solution as per manufacturer guidelines, absorbance measurements were taken at a 550 nm wavelength using a microplate reader. Nitric oxide concentrations were subsequently calculated based on the NaNO_2_ reference curve.

### NIR-triggered NO release

For the investigation of light-controlled NO release, PBS solutions containing UCM-BNN and Gel@UCM-BNN (1 mg/mL) were exposed to intermittent 808 nm NIR irradiation (1.5 W/cm^2^) using a 10-min on/off cycle. At each 10-min interval following the initial exposure, the supernatant was harvested and subjected to nitric oxide quantification. Triplicate measurements were conducted for all experimental groups to maintain data reliability.

### 3T3-L1 preadipocytes differentiation into adipocytes assay

The differentiation potential of 3T3-L1 preadipocytes into mature adipocytes was evaluated through specialized cellular assays. Mouse embryonic fibroblast cells (3T3-L1) were maintained in standard culture environments. Upon reaching 80–90% cell density, enzymatic dissociation using trypsin was performed followed by cell counting. The cells were then plated in 6-well culture dishes at a density of 2–3 × 10^4^ cells per square centimeter, with each well receiving 2 mL of full growth medium. The uniformly distributed 3T3-L1 cultures were maintained at 37°C in a humidified incubator with 5% carbon dioxide. For preparation of differentiation solutions: Solution A was formulated by supplementing 50 mL of high-glucose DMEM (containing 10% FBS and 1% penicillin-streptomycin) with 50 μL aliquots of four components: 1 mM dexamethasone, 500 mM IBMX, 2 mM rosiglitazone and 10 mg/mL insulin. Solution B consisted of the identical basal medium (50 mL) supplemented solely with 50 μL of 10 mg/mL insulin. When cellular confluence approached 90–95%, the existing culture medium was carefully removed from each well. Subsequently, 2 mL of adipogenic differentiation medium (Solution A) was introduced to initiate the differentiation process in the 6-well plates. Following a 48-h exposure to Solution A, the induction medium in the 6-well plate was carefully removed, after which each well received 2 mL of Solution B from the 3T3-L1 adipogenic induction and differentiation medium for a 24-h maintenance period. The experimental protocol involved alternating treatments, with cells exposed to Solution A for 48-h intervals followed by Solution B for 24 h periods in a repeating sequence. Following the treatment regimen, cellular samples were processed using oil red O staining for visualization.

### Intracellular NO release assay

The production of nitric oxide (NO) within cells following NIR exposure was assessed using the fluorescent indicator DAF-FM DA. Adipocytes were plated in 24-well culture dishes at a concentration of 200 000 cells per well and maintained for 1 day in RPMI 1640 growth medium containing 10% FBS and 1% antibiotic solution at 37°C with 5% carbon dioxide. The cellular samples were then treated with DAF-FM DA solution (5 μM concentration in phosphate-buffered saline) for 60 min, after which they were carefully rinsed with PBS to eliminate unbound probe molecules. Next, either UCM-BNN or Gel@UCM-BNN (200 μg/mL concentration) was introduced to the cell cultures and left to interact for 2 hr. Following PBS washes to remove nonadherent compounds, the treated cells underwent irradiation with an 808 nm NIR laser (1.5 W/cm^2^ intensity) for a duration of 10 min. For nuclear visualization, Hoechst 33342 staining was performed for 10 min before capturing fluorescence micrographs using an inverted fluorescence imaging system.

### Cytotoxicity assay

The biocompatibility assessment was conducted on adipocytes and human umbilical vein endothelial cells (HUVECs) employing the CCK-8 method. Cellular suspensions were plated in 96-well culture dishes with an initial concentration of 2 × 10^3^ cells per well. These were maintained for 12 h in RPMI 1640 growth medium enriched with 10% fetal bovine serum and 1% penicillin-streptomycin antibiotic mixture, incubated at 37°C in a humidified atmosphere containing 5% carbon dioxide. Following cellular adherence, the original medium was exchanged with a fresh solution containing different concentrations of Gel@UCM-BNN composite material. The cultures were then grown for periods of 1, 3 and 5 days. In specific experimental conditions, the cells received 808 nm NIR radiation (1.5 W/cm^2^ intensity) for a duration of 10 min. At predetermined intervals, the culture medium was refreshed with a solution containing 10% CCK-8 detection reagent and allowed to react for 2 h. The optical density at a 450 nm wavelength was quantified using a microplate spectrophotometer. Cellular survival rates were determined using the following formula:


$$\text{Cell}\;\text{viability}\;\left(\%\right)\;=\frac{（OD_{e}-OD_{b}）}{（OD_{c}-OD_{b}）}\times 100\%,$$

where OD_e_, OD_b_ and OD_c_ correspond to the measured absorbance values of the test samples, blank controls and negative controls, respectively.

### Live/dead cell staining

HUVECs and adipocytes were plated in 24-well culture dishes with an initial seeding density of 2 × 10^5^ cells per well. These cells were maintained for 12 h in RPMI 1640 culture medium enriched with 10% fetal bovine serum and 1% penicillin-streptomycin antibiotic mixture, incubated at 37°C in a humidified atmosphere containing 5% carbon dioxide. Following this initial culture period, the medium was exchanged with a fresh solution containing different concentrations of Gel@UCM-BNN material. After 24 h of additional incubation, cellular viability was examined through dual staining with Calcein-AM and propidium iodide (PI). Fluorescent microscopy was performed using an inverted microscope system to capture cellular images.

### *In vitro* safety evaluation of 808 nm and 980 nm NIR light

The assessment of cell survival rates was conducted using both adipocytes and HUVECs. These cellular samples were initially placed in 6-well culture dishes with a concentration of 100 000 cells per well and maintained in standard incubation environments for 12 h. After this period, the cells underwent treatment with NIR light at wavelengths of either 808 or 980 nm, with an energy output of 1.5 W/cm^2^ applied for 15 min. Post-treatment procedures involved staining the cellular specimens with Calcein-AM solution (2 μM concentration) and propidium iodide (8 μM concentration) for a duration of 45 min. The distribution patterns of viable versus nonviable cells were then examined through fluorescence microscopy imaging techniques.

### Hemolysis assay

The blood compatibility assessment was conducted through a standardized hemolysis test. In this procedure, 2 mL of venous blood was obtained from male C57BL/6 mice aged 8 weeks. This blood sample underwent centrifugation at 1500 revolutions per minute for 3 min to separate erythrocytes. The resulting red blood cell sediment was cleansed with isotonic saline solution before being reconstituted to achieve a 2 mL suspension. During the experimental process, 100 μL of this erythrocyte suspension was combined with 100 μL of Gel@UCM–BNN solutions at different concentrations, all diluted in 1 mL of physiological saline. A baseline control was established using saline solution without any test material. All test specimens were maintained at 37°C for a duration of 2 h. After this incubation period, the solutions were subjected to centrifugation and the optical density of the resulting supernatant was determined at a 540 nm wavelength using a microplate analyzer. The percentage of hemolysis was computed using the following formula:


$$\text{Hemolysis}\;\text{rate}\left(\%\right)=\left[\left(A_{1}-{\;A}_{2}\right)/\left(A_{3}-{\;A}_{2}\right)\right]\times 100\%,$$

where A_1,_ A_2_ and A_3_ represent the absorbance of the experimental, negative control and positive control groups, respectively.

### Wound healing assay

The evaluation of cellular movement was conducted through a scratch-wound healing experiment. Initially, cells were plated in 24-well culture dishes at a concentration of 50 000 cells per well and grown until they formed a complete monolayer. Straight-line wounds were created with a 200-microliter pipette tip, followed by three PBS washes to eliminate dislodged cells. To reduce cell division effects, medium without serum was introduced. The experimental groups received Gel@UCM–BNN treatment at a concentration of 200 micrograms per milliliter and were exposed to 808 nm NIR light as specified. Wound healing progress was documented at 0, 12 and 24 h intervals using an inverted microscope. The width of the wound at each observation time was measured with ImageJ software, and the migration rate was determined by comparing the percentage of wound closure to the original wound size.

### Transwell assay

The assessment of cellular movement was conducted through a Transwell migration experiment. A 24-well plate setup was employed, with Transwell chambers positioned inside each well. The lower compartment received 500 μL of growth medium, while the upper chamber’s porous membrane was initially moistened with medium lacking serum. Subsequently, a cell suspension (400 μL at a density of 3 × 10^5^ cells/mL) was introduced into the upper compartment. Following a 24-h incubation period under controlled environmental conditions, nonmigratory cells were eliminated from the upper surface. The successfully migrated cells were then preserved through fixation, subjected to staining procedures and visualized under microscopic examination. For quantitative analysis, a minimum of three arbitrary microscopic areas per experimental condition were examined, with cell counts performed utilizing the ImageJ analysis program.

### Tube formation assay

The ability of HUVECs to form blood vessels was evaluated through a standardized tube formation test. Initially, 24-well culture plates were chilled, while Matrigel basement membrane matrix was defrosted at 4°C. Under low-temperature conditions to avoid early solidification, 50 μL of Matrigel was carefully dispensed into each well. The plates were then maintained at 37°C for half an hour to ensure thorough gel formation. Subsequently, HUVECs suspensions were plated onto the Matrigel-coated surfaces at a concentration of 150 000 cells per well, using nutrient-rich culture medium. Experimental groups received Gel@UCM-BNN treatment (200 μg/mL) for 6 hr before undergoing NIR light exposure (808 nm wavelength, 1.5 W/cm^2^ intensity, 15 min duration). Parallel control experiments consisted of untreated cell samples and those subjected solely to NIR radiation. Cellular network formation was monitored with optical microscopy, with digital images recorded for subsequent measurement. For each experimental condition, a minimum of three randomly chosen microscopic fields were examined, with both branching points and cumulative tube dimensions being measured through ImageJ analysis software.

### *In vivo* animal experiment

All experimental protocols were implemented under the oversight and review of the Ethics Committee of Nanchang University, and official approval for the study has been obtained from this committee (NCULAE-20250524001). Male C57BL/6 mice aged eight weeks (body weight: 22–25 g) were housed under specific pathogen-free conditions with a 12-h light/dark cycle and allowed to acclimate for one week prior to experimentation. The animals were divided into five experimental cohorts (*n* = 15 per group): control, NIR irradiation, Gel@UCM-BNN, Gel@UCM + NIR and Gel@UCM-BNN + NIR. Each group consisted of 15 mice, and the hair on the top of the head was shaved off. The mice were anesthetized by inhalation of ether. Autologous adipose tissue was harvested from the inguinal region, minced and rinsed with PBS. Standardized 120 mg (0.2 mL) fat grafts were prepared and then subjected to injection transplantation: using an 18 G sterile needle and a 1 mL sterile syringe, the pre-prepared mixture of materials and adipose tissue (material to adipose tissue ratio of 2:8) was subcutaneously injected into the cranial region of the mice. For the control group, a mixture of adipose tissue and 0.05 mL normal saline was injected; for the NIR group, only 808 nm NIR light irradiation was performed after adipose tissue injection; for the Gel@UCM-BNN group, a mixture of adipose tissue and 0.05 mL Gel@UCM-BNN hydrogel was injected without 808 nm NIR light irradiation after injection; for the Gel@UCM + NIR group, a mixture of adipose tissue and 0.05 mL Gel@UCM hydrogel was injected, followed by irradiation with 808 nm NIR light at a power density of 1.5 W/cm^2^ for a duration of 10 min; for the Gel@UCM-BNN + NIR group, a mixture of adipose tissue and 0.05 mL Gel@UCM-BNN hydrogel was injected, followed by irradiation with 808 nm NIR light at a power density of 1.5 W/cm^2^ for a duration of 10 min. Throughout the entire first week, NIR irradiation was performed on Days 0, 2, 4 and 6 post-transplantation. This 1-week treatment window was specifically chosen to cover the critical early revascularization phase of the fat grafts, which is essential for overcoming ischemia and improving long-term survival. The entire process was strictly followed by sterile operation procedures. During the experiment, the mice showed no abnormalities in diet or behavior, no experimental mice died and no adverse reactions such as inflammation, ulceration or bleeding were observed at the transplantation site. After surgery, all mice received standard postoperative care and were closely monitored to ensure their rapid recovery. Samples were harvested at 1, 4 and 12 weeks post-transplantation for subsequent experimental procedures.

### RNA extraction and sequencing data quality control

Total RNA was isolated following the guidelines provided with the TRIzol reagent. The NanoDrop 2000 spectrophotometer was employed to measure RNA concentration and assess purity, while RNA integrity was evaluated using the Agilent 2100 Bioanalyzer. Library construction for sequencing was carried out with the VAHTS Universal V6 RNA-seq Library Preparation Kit, adhering to established procedures. A third-party service provider conducted high-throughput RNA sequencing and subsequent bioinformatics analyses.

### RNA sequencing and differential expression gene analysis

The sequencing process was carried out on an Illumina NovaSeq 6000 system, producing paired-end reads with a length of 150 base pairs. For identifying differentially expressed genes (DEGs), the DESeq2 package was employed, applying thresholds of q-value below 0.05 and fold change exceeding 2. To investigate biological significance, functional enrichment studies were performed through Gene Ontology (GO) classification and KEGG pathway assessment, utilizing the hypergeometric distribution method to detect statistically overrepresented functional categories. The R programming environment was used to create visual representations of the enriched terms. Furthermore, gene set enrichment analysis (GSEA) was implemented to examine the collective overrepresentation of predefined gene collections. This involved ordering genes by their expression level variations across experimental conditions and determining enrichment significance through analysis of their positional distribution in the ordered sequence.

### Histological examination

Tissue specimens, including adipose tissue collected at 1, 4 and 12 weeks intervals, together with vital organs (cardiac tissue, hepatic tissue, splenic tissue, pulmonary tissue and renal tissue), were preserved in 10% neutral buffered formalin solution at 4°C for a duration of 24 h. Following the fixation process, all specimens underwent paraffin embedding and were sliced into 5 μm sections. For adipose tissue analysis, three distinct histological techniques were employed: hematoxylin-eosin staining, Masson’s trichrome staining and CD31/perilipin dual immunofluorescence staining. Conversely, sections from major organs exclusively received hematoxylin-eosin staining to evaluate systemic biocompatibility. Staining outcomes were subjected to both quantitative and semi-quantitative evaluation utilizing ImageJ analysis software.

### Blood cell analysis

Experimental mice were randomly chosen from both the control cohort and the Gel@UCM-BNN + NIR treatment group (with a minimum of three animals per group). Blood samples (approximately 1 mL in volume) were collected through the orbital bleeding method, and subsequent blood cell analysis was performed.

### Statistical analysis

The results are displayed as mean values accompanied by their standard deviations. For statistical evaluation, GraphPad Prism software (version 7.0) was employed, utilizing either one-way ANOVA or *t*-tests depending on the experimental requirements. Statistical significance was defined as *P*-values below 0.05. Different symbols were used to denote varying significance thresholds: * for *P* < 0.05, ** for *P* < 0.01, *** for *P* < 0.001 and **** for *P* < 0.0001.

## Results and discussion

### Synthesis and characterization of UCM-BNN

**Figure 2:**
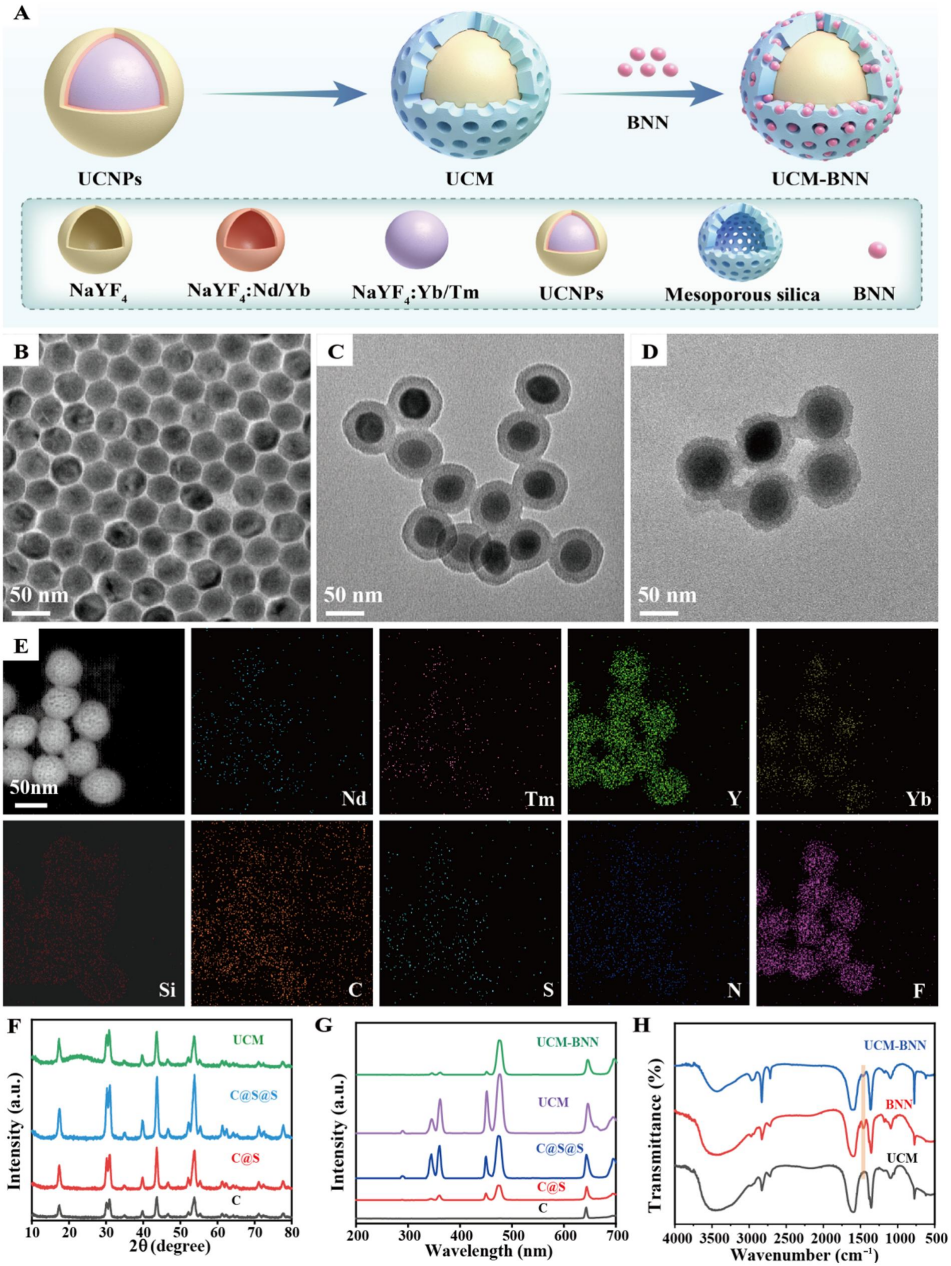
Synthesis and characterization of UCM-BNN nanoparticles. (**A**) Schematic illustration of UCM-BNN synthesis. (**B**–**D**) TEM images of UCNPs, UCM and UCM-BNN (left to right). (**E**) Elemental mapping corresponding to the HAADF-STEM image of UCM-BNN. (**F**) XRD patterns of UCM, core@shell@shell UCNPs, core@shell UCNPs and core UCNPs (top to bottom). (**G**) UCL spectra of UCM-BNN, UCM, core@shell@shell UCNPs, core@shell UCNPs and core UCNPs (top to bottom). (**H**) FTIR spectra of UCM-BNN, BNN and UCM (top to bottom).

The NO-releasing gas therapeutic nanoplatform based on 808 nm NIR-triggered UCNPs was prepared and characterized (Figure 2A). Initially, we synthesized core@shell@shell(C@S@S) structured UCNPs via a thermal decomposition method (Figure 2B and Supplementary Figure S1). The average diameters of the prepared hexagonal phase core, core@shell and core@shell@shell UCNPs were measured to be about 26 nm, 30 nm and 34 nm, respectively (Supplementary Figure S2A–C). To enable the surfaces of these nanoparticles to load NO donors, a layer of mesoporous silica was coated on the UCNPs to form UCM with a particle size of approximately 70 nm (Figure 2C and Supplementary Figure S2D). Subsequently, to confirm that UCM possesses loading capacity and that the surface has pores after silica modification to ensure drug loading efficiency, this was verified by N_2_ adsorption-desorption experiments (Supplementary Figure S3A and B). The NO donor BNN was synthesized as previously described, and its successful synthesis was confirmed by FTIR. In addition, the photo-responsiveness of the synthesized BNN to UV light was confirmed. As shown in Supplementary Figure S4A, the solution of BNN in dimethyl sulfoxide (DMSO) changed from pale yellow to red brown after irradiation with UV light (365 nm), indicating that BNN underwent photochemical degradation to generate NO. The UV–Visible spectrum of BNN revealed a broad UV absorption peak in the wavelength range of 200–400 nm (Supplementary Figure S4B, yellow line), particularly at ∼270 nm. Subsequently, BNN was loaded into the mesopores of UCM to form UCM-BNN. A BNN layer around UCM could be observed under transmission electron microscopy (Figure 2D), and the elemental mapping of UCM-BNN (Figure 2E) also confirmed the successful preparation of UCM-BNN. The XRD patterns (Figure 2F) of UCNPs and UCM are shown. All diffraction peaks can be attributed to β-NaYF_4_ (JCPDS No. 016-0334), and a broad peak of amorphous SiO_2_ from mesoporous silica nanoparticles (MSN) at 2θ = 22° was observed. In addition, the upconversion luminescence (UCL) spectra of UCNPs and UCM were investigated (Figure 2G), and it was found that the UCL intensity increased significantly after coating with the Nd^3+^ shell and the inert NaYF_4_ shell (Figure 2G), enhancing the platform’s efficacy for drug and NO release. Meanwhile, we also used FTIR to detect the functional groups in the products, and a new peak at 1555 cm^−1^ corresponding to the asymmetric bending vibration of N-H bond (Figure 2H), further confirmed the successful BNN loading on UCM.

### Synthesis and characterization of Gel@UCM-BNN hydrogel

**Figure 3:**
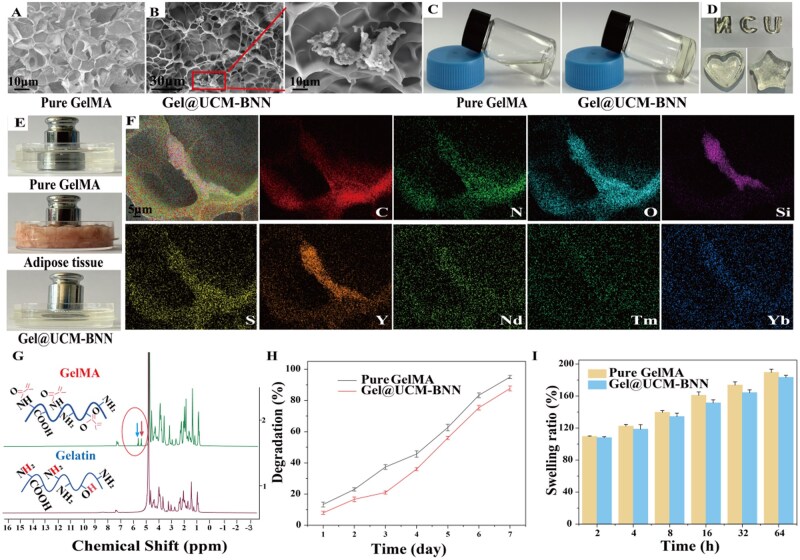
Synthesis and characterization of GelMA hydrogel encapsulating UCM-BNN nanoparticles (Gel@UCM-BNN hydrogel). (**A**) SEM image of GelMA hydrogel. (**B**) SEM images of Gel@UCM-BNN hydrogel. (**C**) *In vitro* cross-linking of GelMA and Gel@UCM-BNN under NIR irradiation. (**D**) Synthesized hydrogels with different shapes. (**E**) Load-bearing capacity of GelMA, adipose tissue and Gel@UCM-BNN hydrogel. (**F**) Elemental mapping corresponding to the HAADF-STEM image of Gel@UCM-BNN hydrogel. (**G**) ^1^H NMR spectra of gelatin and GelMA (blue arrow: H_a_ peak; red arrow: H_b_ peak). (**H**) Degradation ratio of GelMA and Gel@UCM-BNN hydrogel over time. (**I**) Swelling ratio of GelMA and Gel@UCM-BNN hydrogel. Data are presented as mean ± standard deviation (*n* ≥ 3).

First, pure GelMA hydrogels were synthesized *via* the water bath method. After freeze-drying, the cross-sections of the GelMA hydrogels were observed using an SEM, which revealed a relatively uniform porous structure (Figure 3A). To construct a NO-releasing platform for precise filling, we loaded UCM-BNN into the GelMA matrix. SEM imaging showed nanoparticles dispersed within the hydrogel pores (Figure 3B). In addition, to verify the photocurability of the hydrogel *in vitro*. Under NIR irradiation, GelMA without UCM-BNN remained liquid, whereas Gel@UCM-BNN formed a stable gel (Figure 3C), confirming that UCM-BNN enables NIR-triggered crosslinking. Owing to the aforementioned properties, the Gel@UCM-BNN hydrogel exhibits plasticity after curing and can be shaped into various forms using different molds (Figure 3D). To verify its load-bearing capacity, *in vitro* load-bearing experiments were conducted using pure GelMA, adipose tissue and Gel@UCM-BNN hydrogel, respectively. These results indicated that the Gel@UCM-BNN hydrogel exhibited certain mechanical properties (Figure 3E). We also tested the rheological properties and stress–strain curves of pure GelMA, Gel@UCM-BNN hydrogel and adipose tissue, the results (Supplementary Figure S6) show that the mechanical properties of pure GelMA, Gel@UCM-BNN composite hydrogel and adipose tissue are similar. Meanwhile, elemental analysis further verified the successful preparation of Gel@UCM-BNN (Figure 3F). As shown in Figure 3G, the successful conjugation of methacrylate groups with gelatin was confirmed by the presence of peaks at 5.34 and 5.58 ppm in the ^1^H NMR spectrum, respectively. Furthermore, swelling and degradation behaviors of Gel@UCM-BNN were comparable to those of the control group, showing no significant differences (Figure 3H and I).

### *In vitro* evaluation of NO-releasing property

**Figure 4:**
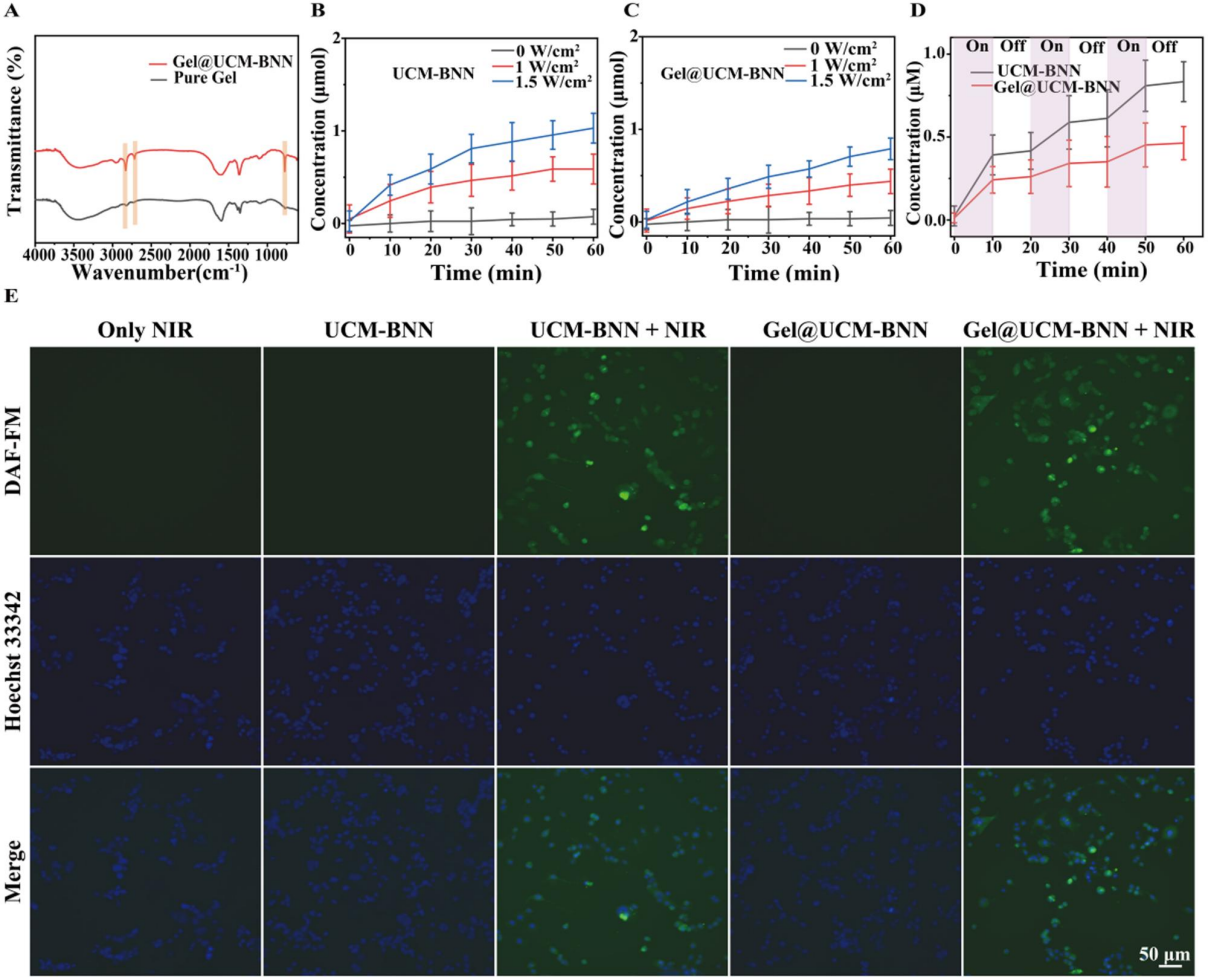
*In vitro* evaluation of NO release performance. (**A**) FTIR spectra of GelMA and Gel@UCM-BNN hydrogel. (**B**, **C**) Evaluation of NO release capacity of UCM-BNN and Gel@UCM-BNN hydrogel (1 mg/mL) excited by 808 nm NIR light under different power densities. (**D**) On/off behavior of NO release from UCM-BNN and Gel@UCM-BNN hydrogel excited by 808 nm NIR light irradiation. (**E**) Fluorescence imaging of adipocytes treated with UCM-BNN and Gel@UCM-BNN hydrogel using Hoechst 33342 and NO fluorescent probe (DAF-FM DA) under dark conditions or after 808 nm NIR light irradiation. Data are presented as mean ± standard deviation (*n* ≥ 3).

The successful synthesis of Gel@UCM-BNN hydrogel was confirmed by both SEM and FTIR spectroscopy. As shown in Figure 4A, the presence of higher peaks at 774 cm^−1^, 2715 cm^−1^ and 2830 cm^−1^ confirms the successful synthesis of Gel@UCM-BNN. After successful preparation, the ability of UCM-BNN and Gel@UCM-BNN to trigger NO release from BNN was investigated. First, the NO release performance of UCM-BNN and Gel@UCM-BNN was evaluated under 808 nm NIR irradiation. Both materials exhibited irradiation power-dependent NO release, though Gel@UCM-BNN showed a slower release rate than UCM-BNN (Figure 4B and C). The platform also displayed excellent photocontrolled release behavior, with NO generation effectively triggered only during NIR irradiation cycles (Figure 4D). These results indicate that the controlled release of NO can be achieved by adjusting the output power and irradiation time of the 808 nm NIR laser. An appropriate concentration of NO is beneficial for promoting angiogenesis [34, 35]. Therefore, this on-demand release performance is conducive to its further biological applications *in vivo*. To investigate the NO release capacity *in vivo*, we designed an intracellular NIR-triggered NO release experiment. 4-Amino-5-methylamino-2',7'-difluorofluorescein diacetate (DAF-FM DA) was used as a fluorescent probe and co-incubated with adipocytes to detect NO release. First, the induced 3T3-L1 cells were detected by Oil Red O staining to verify the successful induction of adipocytes (Supplementary Figure S7). The NO-specific fluorescent probe DAF-FM DA can easily penetrate the cell membrane to enter living cells and then be rapidly catalyzed to form DAF-FM, which cannot penetrate the cell membrane. DAF-FM can react with NO to produce intense green fluorescence, which can finally be observed under a fluorescence microscope. Meanwhile, Hoechst 33342 was used to label cell nuclei, which emits blue fluorescence for cell localization. As shown in Figure 4E, green fluorescence could only be detected in adipocytes when UCM-BNN or Gel@UCM-BNN was present together with NIR irradiation, and the fluorescence intensity of UCM-BNN was higher than that of Gel@UCM-BNN. Quantitative analysis of intracellular NO release in adipocytes (Supplementary Figure S8) further confirms the above results. These findings confirm that NIR irradiation effectively triggers on-demand NO release in living cells and that GelMA encapsulation enables a slower, more sustained release profile.

### *In vitro* biocompatibility and NIR safety evaluation of Gel@UCM-BNN

**Figure 5:**
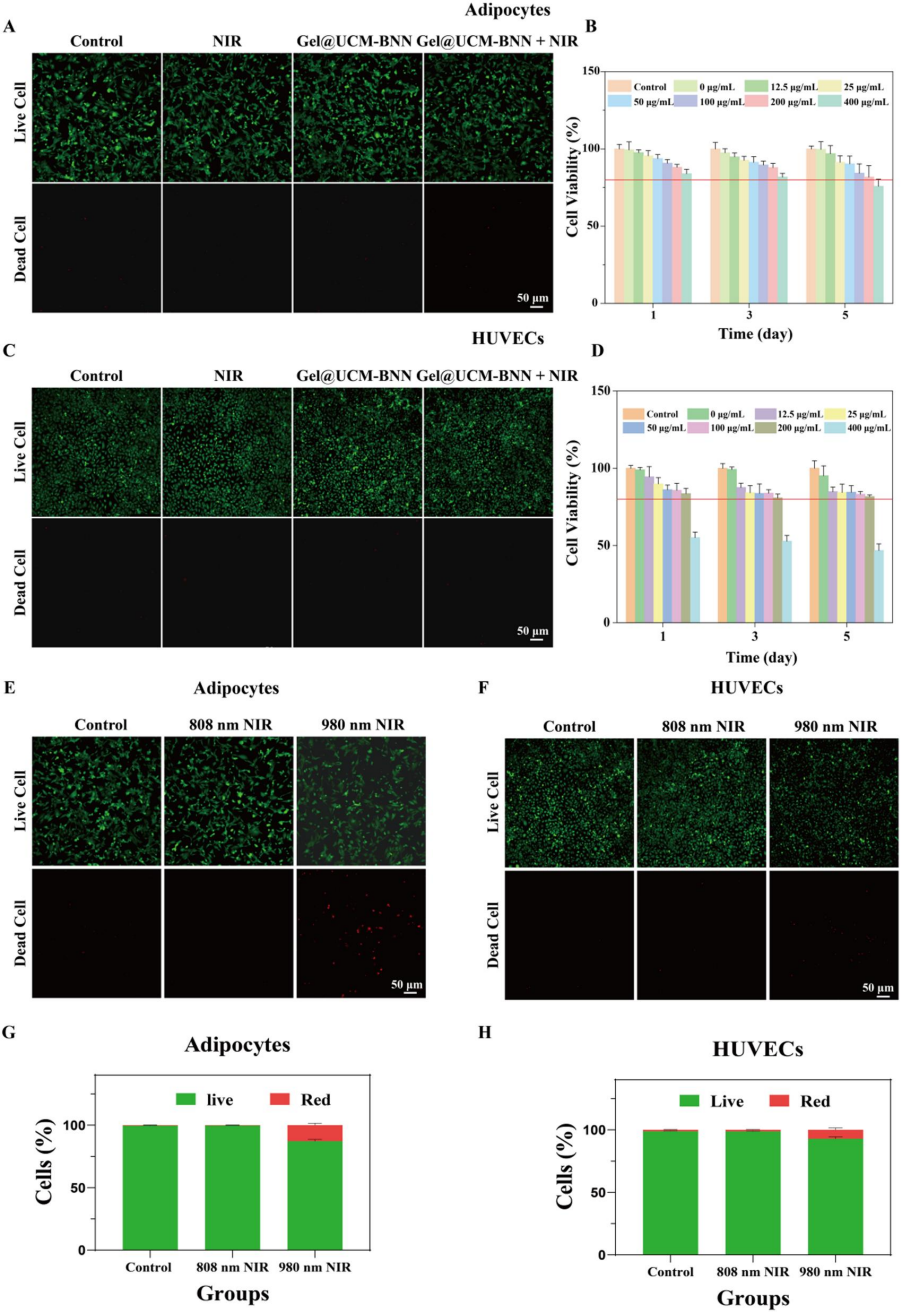
*In vitro* evaluation of cytocompatibility. (**A**) Live/dead staining images of adipocytes treated with different methods. (**B**) Effects of Gel@UCM-BNN hydrogel at different concentrations on the viability of adipocytes at different time points. (**C**) Live/dead staining images of HUVECs treated with different methods. (**D**) Effects of Gel@UCM-BNN hydrogel at different concentrations on the viability of HUVECs cells at different time points. (**E**) Live/dead staining images of adipocytes irradiated with NIR light of different wavelengths. (**F**) Live/dead staining images of HUVECs cells irradiated with NIR light of different wavelengths. (**G**) Percentage of live and dead adipocytes after irradiation with NIR light of different wavelengths. (**H**) Percentage of live and dead HUVECs after irradiation with NIR light of different wavelengths. Data are presented as mean ± standard deviation (*n* ≥ 3).

The biosafety of Gel@UCM-BNN hydrogel, as a biomaterial intended for *in vivo* use, is of critical importance. To evaluate the biocompatibility of the prepared Gel@UCM-BNN hydrogel, we tested its cytocompatibility and hemocompatibility. First, we used the cell counting kit-8 (CCK-8) to evaluate the effects of the Gel@UCM-BNN hydrogel on the viability of adipocytes and HUVECs at 1 day, 3 days and 5 days. Both cell types maintained viability above 80% at Gel@UCM-BNN concentrations lower than 200 μg/mL (Figure 5B and D), indicating no significant cytotoxicity and confirming excellent biocompatibility. The live/dead cell staining of adipocytes (Figure 5A) and HUVECs (Figure 5C), as well as the red blood cell hemolysis test (Supplementary Figure S9) also verified its biosafety.

Owing to the photothermal properties of UCNPs, the NIR light irradiation factor was introduced into the experiment, and the photothermal effect of NIR light needed to be considered. Therefore, we tested the *in vitro* biosafety under 808 nm and 980 nm NIR light irradiation. Under 980 nm NIR irradiation, the water temperature rose to approximately 48°C within 3 min, while under 808 nm NIR irradiation, the water temperature increased to 37°C after 15 min (Supplementary Figure S10). Consistently, 980 nm light caused significant cell death in both adipocytes and HUVECs after 15 min of exposure, while 808 nm light showed no adverse effects (Figure 5E–H). In addition, the potential risk of tissue damage caused by the NIR laser was also investigated. The skin on the top of the mice’s heads was selected for testing: under NIR irradiation, the skin temperature rapidly rose to approximately 48°C within 3 min and continued to increase rapidly. In contrast, after 808 nm NIR light irradiation, it took 15 min to reach an epidermal temperature of about 37°C, which was then maintained stably (Supplementary Figure S11). Collectively, these results underscore the high biosafety of Gel@UCM-BNN and the suitability of 808 nm NIR light for *in vivo* applications.

### *In vitro* tubule formation assay evaluation of Gel@UCM-BNN

**Figure 6:**
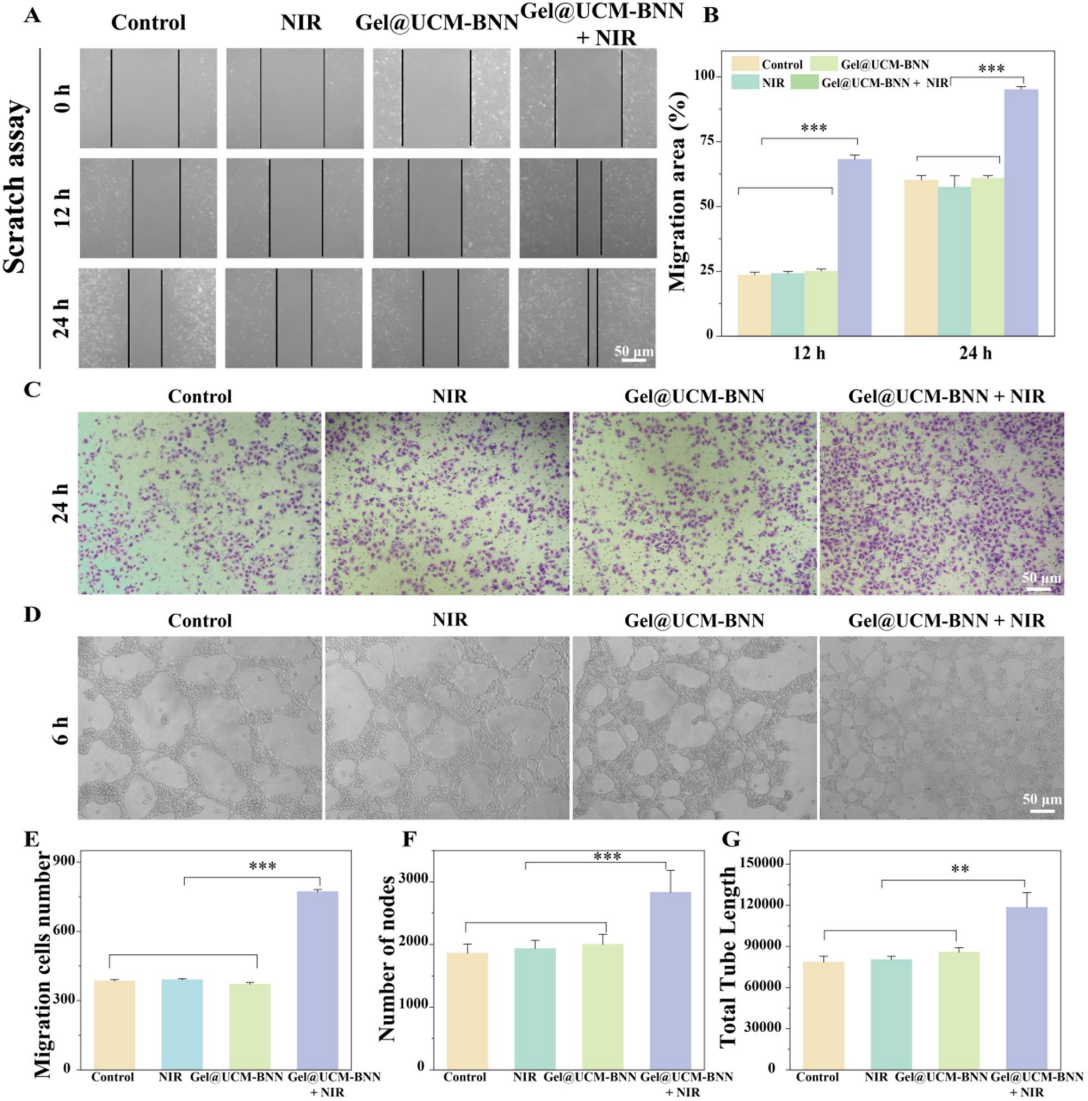
*In vitro* evaluation of NO-induced angiogenesis performance. (**A**) Wound healing assay treated with different methods. (**B**) Quantitative analysis results of the migration area in the wound healing assay. Transwell assay (**C**) and quantitative analysis results (**E**) of the number of migrated cells. (**D**) Evaluation of tube formation ability of HUVECs and quantitative analysis results of vascular node number (**F**) and total tube length (**G**). Data are presented as mean ± standard deviation (*n* ≥ 3; **P < 0.01, ***P < 0.001).

To further evaluate the effect of the Gel@UCM-BNN hydrogel, migration, proliferation and tube formation assays of HUVECs were performed to assess its ability to promote angiogenesis *in vitro*. In the wound healing assay, the Gel@UCM-BNN + NIR group exhibited significantly enhanced HUVEC migration compared to other groups (Figure 6A) with accelerated wound closure quantified in Figure 6B. Similarly, the proliferation assay revealed that Gel@UCM-BNN + NIR substantially promoted HUVEC growth (Figure 6C), an effect further quantified by cell counting (Figure 6E). As shown in Figure 6D, the number of tube formations in the Gel@UCM-BNN + NIR group was significantly higher than that in other groups. Figure 6F and G show the quantitative graphs of the number of nodes and the total tube length, respectively. Collectively, these results demonstrate that NIR-triggered NO release from Gel@UCM-BNN effectively enhances HUVEC migration, proliferation and tube formation, highlighting its pro-angiogenic potential [36].

### Data quality control for RNA sequencing

**Figure 7:**
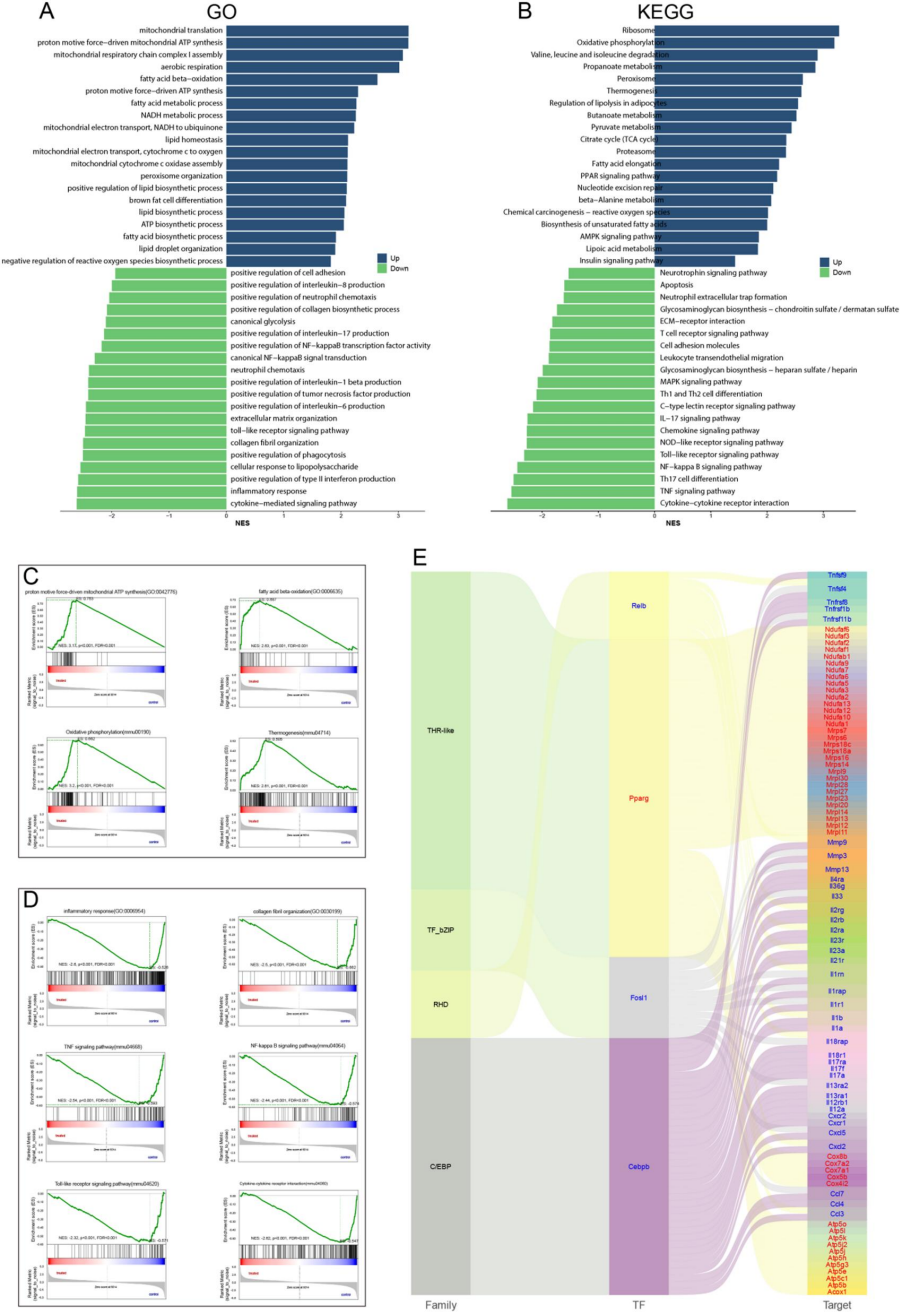
Suppression of inflammatory response and remodeling of energy metabolism in transplanted fat after BNN Gel@UCM-BNN + NIR treatment. (**A**, **B**) Analysis of the GO biological process and KEGG pathway analysis for differentially expressed genes between the Gel@UCM-BNN + NIR group and the control group indicates improved aerobic respiration and suppressed inflammatory response. (**C**) Key upregulated terms involved in energy metabolism. (**D**) Key downregulated terms involved in the inflammatory response. (**E**) Transcription factor analysis indicates that Relb and Pparg serve as critical transcription factors for energy metabolism (highlighted in red) and Fosl1 and Cebpb for inflammatory response (highlighted in blue).

To explore the molecular mechanism of NO in fat transplantation, high-throughput RNA sequencing (RNA-seq) was performed. At 4 weeks after fat transplantation, RNA was extracted from the control group and Gel@UCM-BNN + NIR group for RNA sequencing and bioinformatics analysis. All samples met the quality control standards. As shown in Supplementary Figure S12, compared with the control group, the samples in the Gel@UCM-BNN + NIR group showed significant differences. Gene Ontology (GO) and KEGG pathway analyses indicated that Gel@UCM‑BNN + NIR treatment significantly attenuated inflammatory responses in fat grafts. Among the downregulated differentially expressed genes (DEGs), we observed enrichment in GO terms related to positive regulation of IL‑8, IL‑17, IL‑6, IL‑1β and type II interferon production (Figure 7A–D). Correspondingly, KEGG analysis further highlighted the suppression of multiple key inflammatory pathways, including neutrophil extracellular trap formation, T cell receptor signaling, leukocyte transendothelial migration, Th1 and Th2 cell differentiation, IL-17 signaling, chemokine signaling, NOD-like receptor signaling, Toll-like receptor signaling, NF-κB signaling and TNF signaling pathways.

In contrast, upregulated genes were associated with enhanced aerobic energy metabolism, including ATP synthesis driven by proton motive force and the mitochondrial electron transport chain, oxidative phosphorylation and fatty acid β-oxidation. Furthermore, as illustrated in the mulberry chart (Figure 7E), transcription factor analysis identified Pparg as an upstream regulator of the Nduf, Mrp, Cox and Atp gene families, which are involved in aerobic respiration and energy production. In contrast, Relb, Fosl1 and Cebpb were identified as upstream transcriptional regulators of the Tnf, Mmp, IL, Cxcl and Ccl gene families, which mediate chemotaxis and inflammatory responses.

### *In vivo* animal experiments

**Figure 8:**
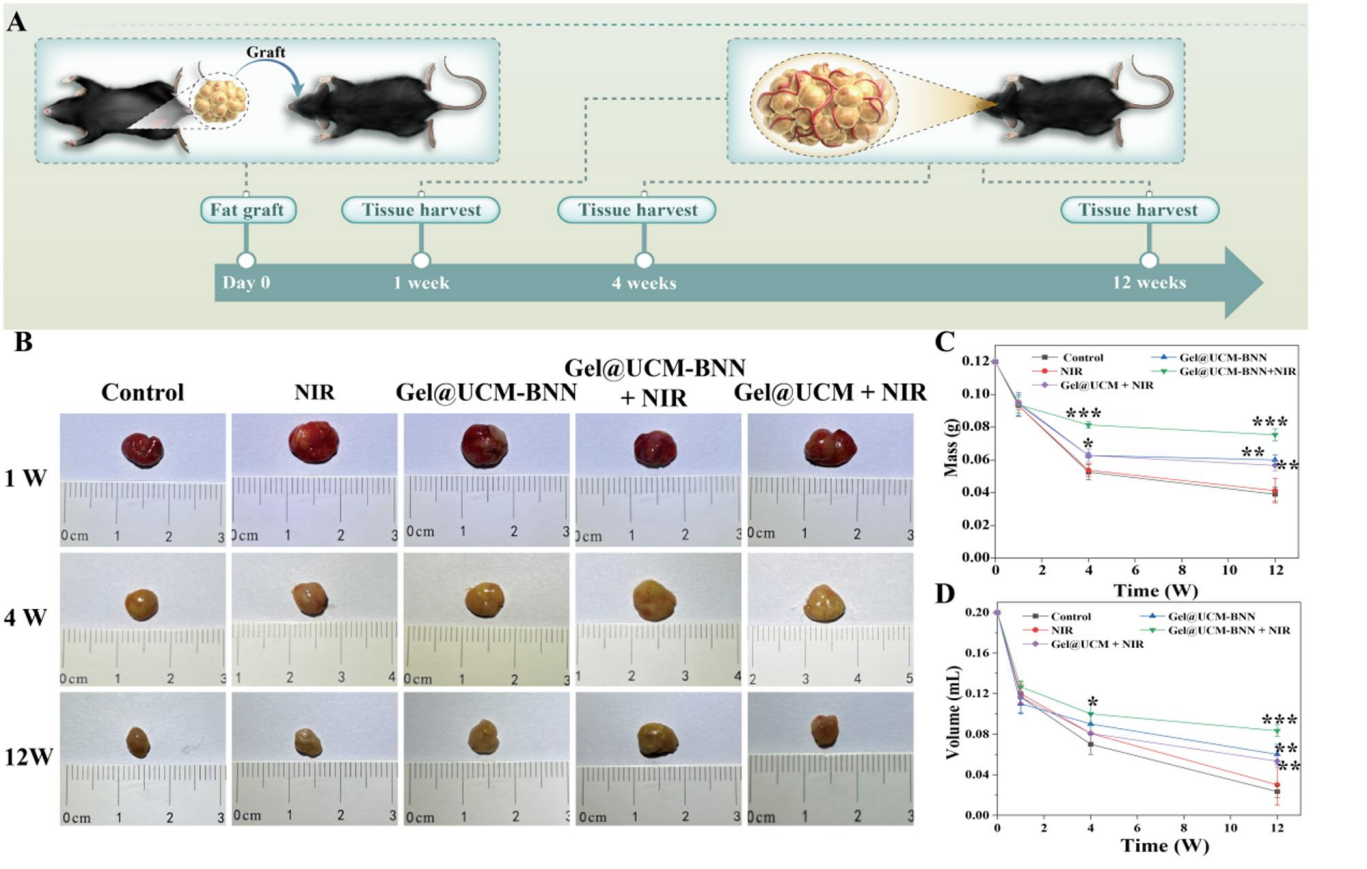
Evaluation of survival of fat grafts in animals. (**A**) Time distribution of sample collection after fat transplantation. (**B**) Gross morphological appearance and changes in weight (**C**) and volume (**D**) of tissues in each group (control group, NIR group, Gel@UCM-BNN group, Gel@UCM-BNN + NIR group, Gel@UCM + NIR group) at different time points (1, 4 and 12 weeks). Data are expressed as mean ± standard deviation (*n* ≥ 3; *P < 0.05, **P < 0.01, ***P < 0.001).

To further observe the effect of NO on adipose tissue, we conducted an *in vivo* study to investigate its role in adipose tissue (Figure 8A). Eight-week-old male C57BL/6 mice were selected and classified into five groups (control group, NIR group, Gel@UCM-BNN group, Gel@UCM-BNN + NIR group and Gel@UCM + NIR group) for fat transplantation. Samples were collected at three time points (1, 4 and 12 weeks) for subsequent observation. The results of graft sampling are shown in Figure 8B: no significant changes were observed at 1 week after transplantation. By 4 and 12 weeks, however, the Gel@UCM-BNN + NIR group displayed clear vascularization on the graft surface, surpassing other groups. The mass (Figure 8C) and volume (Figure 8D) of adipose tissue were measured and recorded. The mass and volume of grafts in all five groups showed a decreasing trend at 1 week, 4 weeks and 12 weeks after transplantation. Nevertheless, at 4 weeks and 12 weeks, the Gel@UCM-BNN group, the Gel@UCM-BNN + NIR group and Gel@UCM + NIR group exhibited increased retention of graft mass and volume compared with the blank group, with the Gel@UCM-BNN + NIR group showing the most significant effect. Ultrasound imaging at 12 weeks further indicated that the Gel@UCM‑BNN + NIR group had the lowest incidence of oil cysts (Supplementary Figure S15), supporting its role in improving graft quality and survival.

### Histological examination

**Figure 9:**
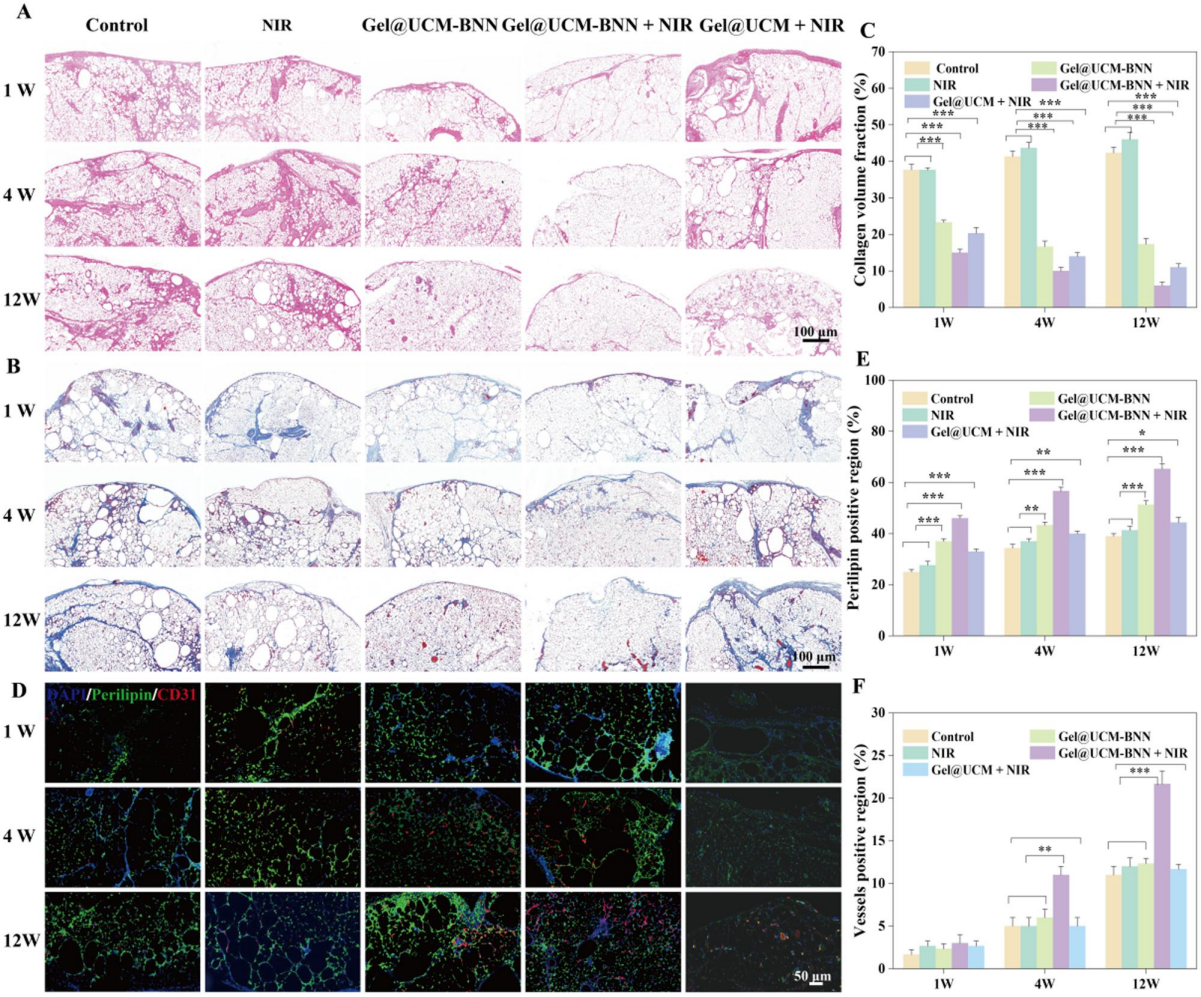
*In vivo* animal experiments. (**A**) HE staining results of samples collected from the control group, NIR group, Gel@UCM-BNN group and Gel@UCM-BNN + NIR group, Gel@UCM + NIR group at 1, 4 and 12 weeks after fat transplantation. (**B**, **C**) Masson staining results of fat grafts and quantitative analysis results of collagen. (**D–F**) Immunofluorescence staining images of different groups collected at 1, 4 and 12 weeks post-transplantation (red: CD31; green: perilipin; blue: DAPI), as well as quantitative analysis results of perilipin-positive areas and quantitative analysis results of vascular regeneration areas. Data are expressed as mean ± standard deviation (*n* ≥ 3; *P < 0.05, **P < 0.01, ***P < 0.001).

To further observe the microstructure of the tissues, we carried out hematoxylin-eosin (H&E) staining on the obtained tissues. As shown in Figure 9A, the results revealed obvious stratification in all five groups of tissues. In the control and NIR groups, significant inflammatory cell infiltration was observed around the grafts at 1 week after transplantation, with vacuole formation in the peripheral area and large acellular structural regions in the innermost layer. This indicated that adipocytes in the control group underwent extensive necrosis due to ischemia and hypoxia in the early stage after transplantation. In comparison with the control group, NIR group and Gel@UCM + NIR group, the Gel@UCM-BNN group showed reduced inflammatory cell infiltration, a small number of vacuoles formed by adipocyte necrosis and relatively higher tissue integrity. The Gel@UCM-BNN + NIR group exhibited a significantly reduced extent of inflammatory infiltration compared with the previous four groups, with fewer vacuoles in the grafts, more uniform adipocyte morphology and more intact tissue structure. At 4 weeks after transplantation, the control group, NIR group and Gel@UCM + NIR group partially showed obvious fused areas after cell necrosis, with eosinophilic acellular regions and fibrosis inside. In comparison with the Control group, NIR group and Gel@UCM + NIR, the Gel@UCM-BNN group had fewer fused areas after cell necrosis. The Gel@UCM-BNN + NIR group exhibited significantly reduced necrotic areas compared with the previous three groups, with intact adipocyte structures. At 12 weeks, the control, NIR groups and Gel@UCM + NIR group presented extensive tissue loss and fibrosis. The Gel@UCM‑BNN group contained only small necrotic foci, whereas the Gel@UCM‑BNN + NIR group showed uniformly distributed viable adipocytes and the highest degree of tissue integrity. In addition, H&E staining of major organs (Supplementary Figure S13) and complete blood count analysis (Supplementary Figure S14) confirmed the *in vivo* biosafety of the hydrogel system.

To observe the tissue fibrosis of the five groups of grafts in the late stage after transplantation, we performed Masson staining on the tissues of each group separately. As illustrated in Figure 9C, the control group, NIR group and Gel@UCM + NIR group exhibited vacuolar changes, with a significant reduction in viable cells inside the tissues, which were composed of thick blue-stained collagen fibers. The Gel@UCM-BNN group also showed fused vacuoles and the formation of blue-stained collagen fibers, while the Gel@UCM-BNN + NIR group displayed more uniformly distributed adipocytes and significantly less collagen deposition, as quantified in Figure 9B.

To compare the microvessel density and adipocyte survival of grafts in the late stage after transplantation and observe the relationship between them, immunofluorescence staining was performed on the obtained tissues of each group. As shown in Figure 9E, red fluorescence labels CD31 (+) blood vessels, green fluorescence labels perilipin (+) viable adipocytes and blue fluorescence labels cell nuclei. The Gel@UCM-BNN + NIR group showed a marked increase in angiogenesis and preserved adipocyte area. Quantitative analysis of five randomly selected fields for perilipin (+) and CD31 (+) areas (Figure 9D and F) confirmed these observations. These results demonstrate that NO promotes graft vascularization, thereby enhancing adipose tissue survival after transplantation.

## Conclusion

Targeting the critical challenges in soft tissue reconstruction, such as unstable survival rates of transplanted fat and poor filling effects, this study drew inspiration from the rapid solidification of ‘Frost Formation’ to design and construct an NIR-responsive NO-controlled-release hydrogel platform, named Gel@UCM-BNN. To confer loading capacity, the surface of UCNPs was modified with a silica layer, followed by the incorporation of the NO donor BNN, resulting in the formation of UCM-BNN. Upon incorporation into a GelMA hydrogel, the composite converts deeply penetrating NIR light into UV emissions *in situ*, simultaneously driving hydrogel photocrosslinking and triggering controlled NO release. This platform can effectively enhance the blood supply microenvironment of adipose tissue after transplantation, thereby enhancing the survival rate of transplanted fat. However, some aspects require further optimization at present: first, the release kinetics of NO should be better aligned with the angiogenesis cycle of transplanted adipose tissue to enhance therapeutic precision; second, the long-term biodegradation *in vivo* and biosafety of Gel@UCM-BNN need to be verified by extending the observation period. Overall, this research not only proposes a new strategy for the treatment of soft tissue depression based on a NIR-responsive NO-controlled release hydrogel platform but also offers experimental references for achieving more precise, safe and long-lasting fat grafting. Furthermore, it lays a solid foundation for future clinical translation in areas such as breast and buttock augmentation, as well as dorsal hand filling.

## Supplementary Material

rbag086_Supplementary_Data

## Data Availability

The data supporting the results of this study are available from the corresponding author upon reasonable request.

## References

[1] Baum SH , Pförtner R, Ladwein F, Schmeling C, Rieger G, Mohr C. Use of dermis-fat grafts in the prevention of Frey’s syndrome after parotidectomy. J Craniomaxillofac Surg 2016;44:301–8. 26805921 10.1016/j.jcms.2015.12.007

[2] Echevarria Cruz EC , Heyer BE, Moenster JM. Treatment of facial asymmetry caused by Parry-Romberg syndrome using fat transfer. Cureus 2025;17:e80977. 40260330 10.7759/cureus.80977 PMC12010110

[3] Xia P , Liu C, Wei X, Guo J, Luo Y. 3D-printed hydrogel scaffolds with drug- and stem cell-laden core/shell filaments for cancer therapy and soft tissue repair. J Mater Chem B 2024;12:11491–501. 39402943 10.1039/d4tb01571a

[4] Davis MJ , Perdanasari AT, Abu-Ghname A, Gonzalez SR, Chamata E, Rammos CK, Winocour SJ. Application of fat grafting in cosmetic breast surgery. Semin Plast Surg 2020;34:24–9. 32071576 10.1055/s-0039-1700958 PMC7023973

[5] Lin Z , Yang K, Li G, Wei S, Liu Y. Efficacy and safety of subcutaneous temporal autologous micro-fat augmentation. Aesthetic Plast Surg 2020;44:2098–106. 32372123 10.1007/s00266-020-01741-y

[6] Onur Erol O , Agaoglu G, Jawad MA. Combined non-ablative laser and microfat grafting for burn scar treatment. Aesthet Surg J 2019;39:NP55–67. 30403775 10.1093/asj/sjy291

[7] Ramot Y , Silyuk T, Murad S, Zlotogorski A. Hirsutism induced by facial autologous fat grafting. Skin Appendage Disord 2020;6:41–3. 32021861 10.1159/000502444 PMC6995947

[8] Chen A , Zhang L, Chen P, Zhang C, Tang S, Chen X. Comparison of the efficacy and safety of cell-assisted lipotransfer and platelet-rich plasma assisted lipotransfer: what should We expect from a systematic review with meta-analysis? Cell Transplant 2021;30:963689721989607. 33845642 10.1177/0963689721989607 PMC8058798

[9] Li H , Li Z, Zhang X, Lin Y, Zhang T, Gan L, Mu D. The effect of exogenous mitochondria in enhancing the survival and volume retention of transplanted fat tissue in a nude mice model. Stem Cell Res Ther 2024;15:321. 39334429 10.1186/s13287-024-03938-3 PMC11438222

[10] Liu L , Wang Q, Liao H, Ye J, Huang J, Li S, Peng H, Yu X, Wen H, Wang X. Soluble microneedle patch with photothermal and NO-release properties for painless and precise treatment of ischemic perforator flaps. J Mater Chem B 2021;9:7725–33. 34586148 10.1039/d1tb00491c

[11] Coneski PN , Schoenfisch MH. Nitric oxide release: part III. Measurement and reporting. Chem Soc Rev 2012;41:3753–8. 22362308 10.1039/c2cs15271a PMC3341472

[12] Garthwaite J. Concepts of neural nitric oxide-mediated transmission. Eur J Neurosci 2008;27:2783–802. 18588525 10.1111/j.1460-9568.2008.06285.x PMC2610389

[13] Schwentker A , Billiar TR. Nitric oxide and wound repair. Surg Clin North Am 2003;83:521–30. 12822723 10.1016/S0039-6109(02)00207-4

[14] Su C-H , Li W-P, Tsao L-C, Wang L-C, Hsu Y-P, Wang W-J, Liao M-C, Lee C-L, Yeh C-S. Enhancing microcirculation on multitriggering manner facilitates angiogenesis and collagen deposition on wound healing by photoreleased NO from hemin-derivatized colloids. ACS Nano 2019;13:4290–301. 30883107 10.1021/acsnano.8b09417

[15] Witte MB , Barbul A. Role of nitric oxide in wound repair. Am J Surg 2002;183:406–12. 11975928 10.1016/s0002-9610(02)00815-2

[16] Sortino S. Light-controlled nitric oxide delivering molecular assemblies. Chem Soc Rev 2010;39:2903–13. 20556272 10.1039/b908663n

[17] Liu S , Sun Y, Zhang T, Cao L, Zhong Z, Cheng H, Wang Q, Qiu Z, Zhou W, Wang X. Upconversion nanoparticles regulated drug & gas dual-effective nanoplatform for the targeting cooperated therapy of thrombus and anticoagulation. Bioact Mater 2022;18:91–103. 35387173 10.1016/j.bioactmat.2022.03.013 PMC8961464

[18] Ye J , Jiang J, Zhou Z, Weng Z, Xu Y, Liu L, Zhang W, Yang Y, Luo J, Wang X. Near-infrared light and upconversion nanoparticle defined nitric oxide-based osteoporosis targeting therapy. ACS Nano 2021;15:13692–702. 34328303 10.1021/acsnano.1c04974

[19] Jiang J , Xie J, Zhou L, Han W, Ye J, Hu D, Xie W, Qiu J, Chen R, Wang X. Near infrared responsive nitric oxide and carbon monoxide nanoplatform for synergistic photodynamic therapy against periodontitis. Chem Eng J 2024;480:147850.

[20] Wang Z , Fu X, Dai C, Yang B, Wang W, Fan C, Zhang P, Sun J, Sun D. NIR II-triggered core-shell upconversion nanocomposites for peroxynitrite-boosted anti-infection against diabetic wound. Chem Eng J 2024;480:148271.

[21] Fan J , He Q, Liu Y, Zhang F, Yang X, Wang Z, Lu N, Fan W, Lin L, Niu G, He N, Song J, Chen X. Light-responsive biodegradable nanomedicine overcomes multidrug resistance via NO-enhanced chemosensitization. ACS Appl Mater Interfaces 2016;8:13804–11. 27213922 10.1021/acsami.6b03737 PMC5233726

[22] Li L , Lin Z, Lu X, Chen C, Xie A, Tang Y, Zhang Z. Photo-controlled and photo-calibrated nanoparticle enabled nitric oxide release for anti-bacterial and anti-biofilm applications. RSC Adv 2022;12:33358–64. 36506481 10.1039/d2ra05352g PMC9686666

[23] Roy E , Wong HY, Villani R, Rouille T, Salik B, Sim SL, Murigneux V, Stark MS, Fink JL, Soyer HP, Walker G, Lyons JG, Saunders N, Khosrotehrani K. Regional variation in epidermal susceptibility to UV-induced carcinogenesis reflects proliferative activity of epidermal progenitors. Cell Rep 2020;31:107702. 32492418 10.1016/j.celrep.2020.107702

[24] Chen X , Zhang D, Wang X, Liu Z, Kang H, Liu C, Chen F. Preparation of porous GelMA microcarriers by microfluidic technology for stem-cell culture. Chem Eng J 2023;477:146444.

[25] Tang S , Huang J, Du S, Feng W, Wang X, Cheng C, Yang T, Ding G. Multifunctional NGF-loaded PLGA microsphere-PCL/GelMA scaffold enables coordinated osteo-neuro-vascular regeneration via PI3K/AKT pathway activation. Chem Eng J 2025;520:165993.

[26] Sun M , Sun X, Wang Z, Guo S, Yu G, Yang H. Synthesis and properties of gelatin methacryloyl (GelMA) hydrogels and their recent applications in load-bearing tissue. Polymers (Basel) 2018;10:1290. 30961215 10.3390/polym10111290 PMC6401825

[27] Zhang Y , Yin P, Huang J, Yang L, Liu Z, Fu D, Hu Z, Huang W, Miao Y. Scalable and high-throughput production of an injectable platelet-rich plasma (PRP)/cell-laden microcarrier/hydrogel composite system for hair follicle tissue engineering. J Nanobiotechnology 2022;20:465. 36329527 10.1186/s12951-022-01671-8 PMC9632161

[28] Jing X , Xu C, Su W, Ding Q, Ye B, Su Y, Yu K, Zeng L, Yang X, Qu Y, Chen K, Sun T, Luo Z, Guo X. Photosensitive and conductive hydrogel induced innerved bone regeneration for infected bone defect repair. Adv Healthc Mater 2023;12:e2201349. 36325633 10.1002/adhm.202201349

[29] Wu H , Shang Y, Sun W, Ouyang X, Zhou W, Lu J, Yang S, Wei W, Yao X, Wang X, Zhang X, Chen Y, He Q, Yang Z, Ouyang H. Seamless and early gap healing of osteochondral defects by autologous mosaicplasty combined with bioactive supramolecular nanofiber-enabled gelatin methacryloyl (BSN-GelMA) hydrogel. Bioact Mater 2023;19:88–102. 35441114 10.1016/j.bioactmat.2022.03.038 PMC9005961

[30] Zhu S , Yu C, Liu N, Zhao M, Chen Z, Liu J, Li G, Huang H, Guo H, Sun T, Chen J, Zhuang J, Zhu P. Injectable conductive gelatin methacrylate/ oxidized dextran hydrogel encapsulating umbilical cord mesenchymal stem cells for myocardial infarction treatment. Bioact Mater 2022;13:119–34. 35224296 10.1016/j.bioactmat.2021.11.011 PMC8844712

[31] Kurian AG , Singh RK, Patel KD, Lee J-H, Kim H-W. Multifunctional GelMA platforms with nanomaterials for advanced tissue therapeutics. Bioact Mater 2022;8:267–95. 34541401 10.1016/j.bioactmat.2021.06.027 PMC8424393

[32] Xiao S , Zhao T, Wang J, Wang C, Du J, Ying L, Lin J, Zhang C, Hu W, Wang L, Xu K. Gelatin methacrylate (GelMA)-based hydrogels for cell transplantation: an effective strategy for tissue engineering. Stem Cell Rev Rep 2019;15:664–79. 31154619 10.1007/s12015-019-09893-4

[33] Li L , Liu Z, Li Y, Dong Y. Frost deposition on a horizontal cryogenic surface in free convection. Int J Heat Mass Transf 2017;113:166–75.

[34] Pelegrino MT , Weller RB, Chen X, Bernardes JS, Seabra AB. Chitosan nanoparticles for nitric oxide delivery in human skin. Medchemcomm 2017;8:713–9. 30108789 10.1039/c6md00502k PMC6072359

[35] Heo T-H , Jang H-J, Jeong G-J, Yoon J-K. Hydrogel-based nitric oxide delivery systems for enhanced wound healing. Gels 2025;11:621. 40868752 10.3390/gels11080621 PMC12385316

[36] Le Thi P , Tran DL, Park KM, Lee S, Oh DH, Park KD. Biocatalytic nitric oxide generating hydrogels with enhanced anti-inflammatory, cell migration, and angiogenic capabilities for wound healing applications. J Mater Chem B 2024;12:1538–49. 38251728 10.1039/d3tb01943h

