# Supplementary material for: A ‘frost formation’-inspired near-infrared-responsive nitric oxide-releasing hydrogel for enhancing fat graft survival
Source: Regen Biomater. 2026 May 5;13:rbag086. doi: 10.1093/rb/rbag086 (PMC13202454; doi:10.1093/rb/rbag086)
Supplement: rbag086_Supplementary_Data [file rbag086_supplementary_data.docx]

**A “Frost Formation”-Inspired Near-Infrared Responsive Nitric Oxide Releasing Hydrogel for Enhancing Fat Graft Survival**

*Yanglong Zhu*^a,b,c^*, Yatian Wei*^d^*, Zixun Lan*^b^*, Yuanzheng Zhu*^a,c^*, Hengyu Wu*^a,c^*, Tingting Zhong*^e^*, Xiang Shen*^b^*, Ganghua Yang*^a,c^*, Yangyan Yi*^a,c^* and *Xiaolei Wang*^b,e^*

^a^ Department of Plastic Surgery, The Second Affiliated Hospital, Jiangxi Medical College, Nanchang University, Nanchang, Jiangxi 330006, P. R. China

^b^ The National Engineering Research Center for Bioengineering Drugs and the Technologies, Institute of Translational Medicine, Nanchang University, Jiangxi, 330088, P. R. China.

^c^ Jiangxi Province Key laboratory of Precision Cell Therapy, Jiangxi Medical College, Nanchang, Jiangxi 330006, P. R. China

^d^ Department of Thoracic surgery, The Second Affiliated Hospital, Jiangxi Medical College, Nanchang University, Nanchang, Jiangxi 330006, P. R. China

^e^ School of Chemistry and Chemical Engineering, Nanchang University, Nanchang, Jiangxi 330088, P. R. China.

*** Corresponding authors’ E-mail addresses:**

[yyy0218@126.com](mailto:yyy0218@126.com) (Yang yan Yi); [wangxiaolei@ncu.edu.cn](mailto:wangxiaolei@ncu.edu.cn) (Xiaolei Wang).

**Figures**

**
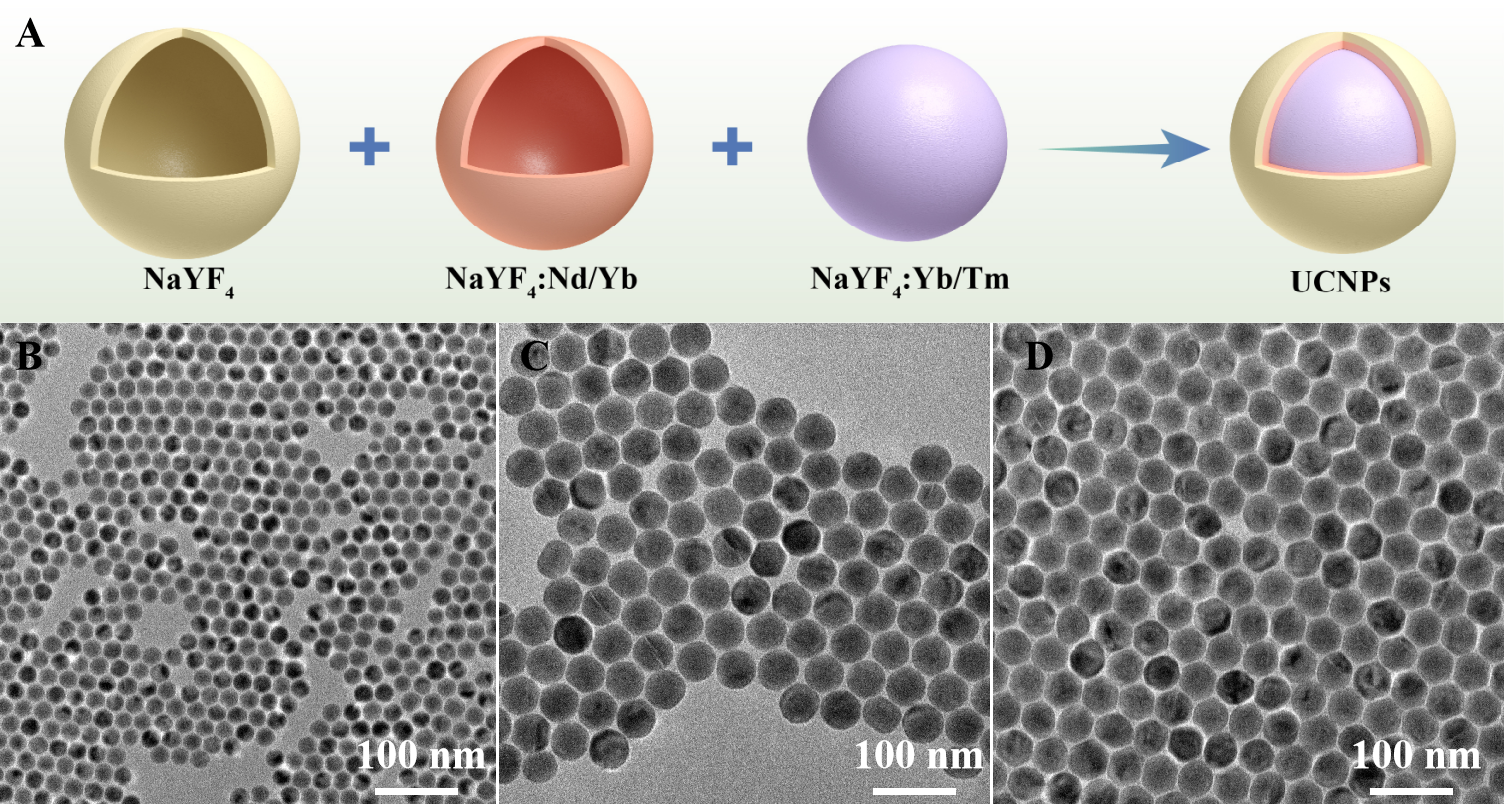
**

**Figure S1.** (A) Synthesis process of UCNPs. (B-D) TEM images of NaYF_4_:Yb/Tm (25/0.5%) core nanocrystals; NaYF_4_:Yb/Tm (25/0.5%) core/NaYF_4_:Nd/Yb (20/10%) shell nanocrystals; and NaYF_4_:Yb/Tm (25/0.5%)core/NaYF_4_:Nd/Yb (20/10%) shell/NaYF₄ shell nanocrystals, respectively.


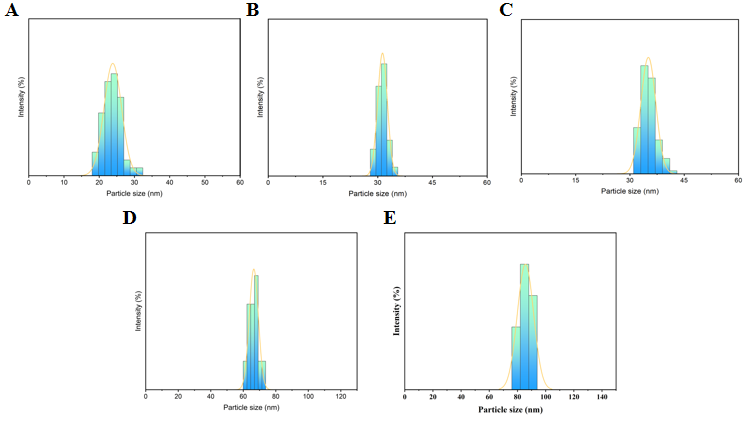


**Figure S2.** (A-E): Particle size analysis of NaYF_4_:25%Yb, 0.5%Tm (core); NaYF_4_:25%Yb, 0.5%Tm@NaYF_4_:20%Nd, 10%Yb (core@shell); NaYF_4_:25%Yb, 0.5%Tm@NaYF_4_:20%Nd, 10%Yb@NaYF_4_ (core@shell@shell); UCNPs@SiO_2_ (UCM) and UCM-BNN.


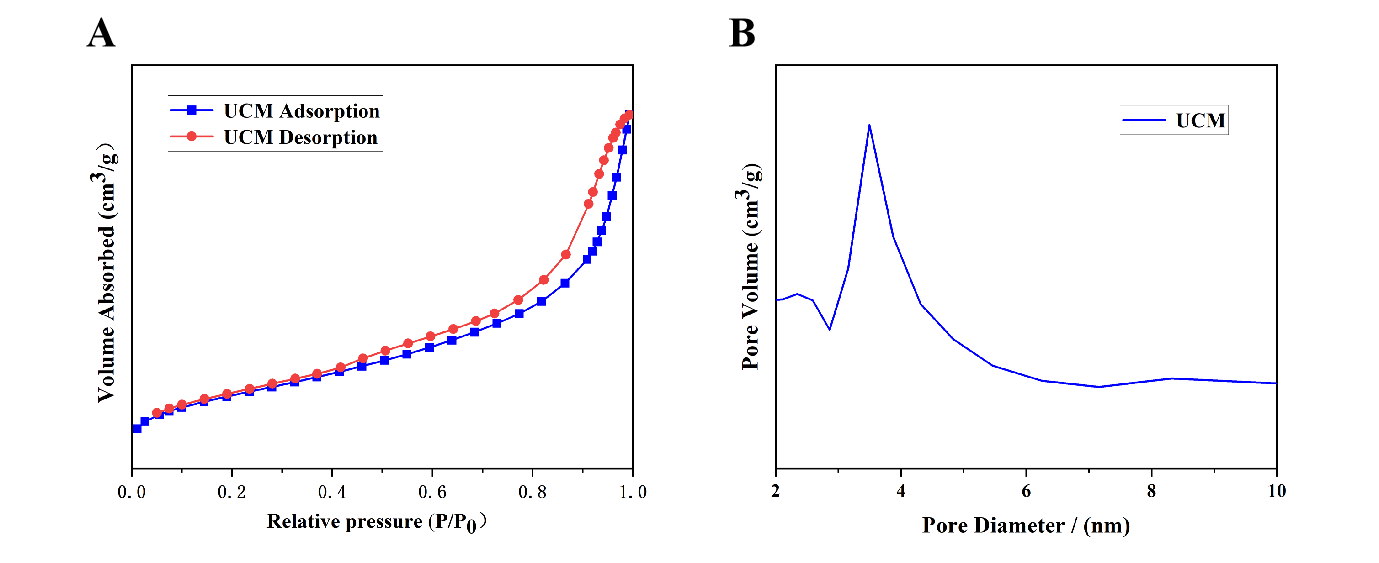


**Figure S3.** (A) N_2_ adsorption-desorption isotherm of UCM; (B) Pore size distribution of UCM.


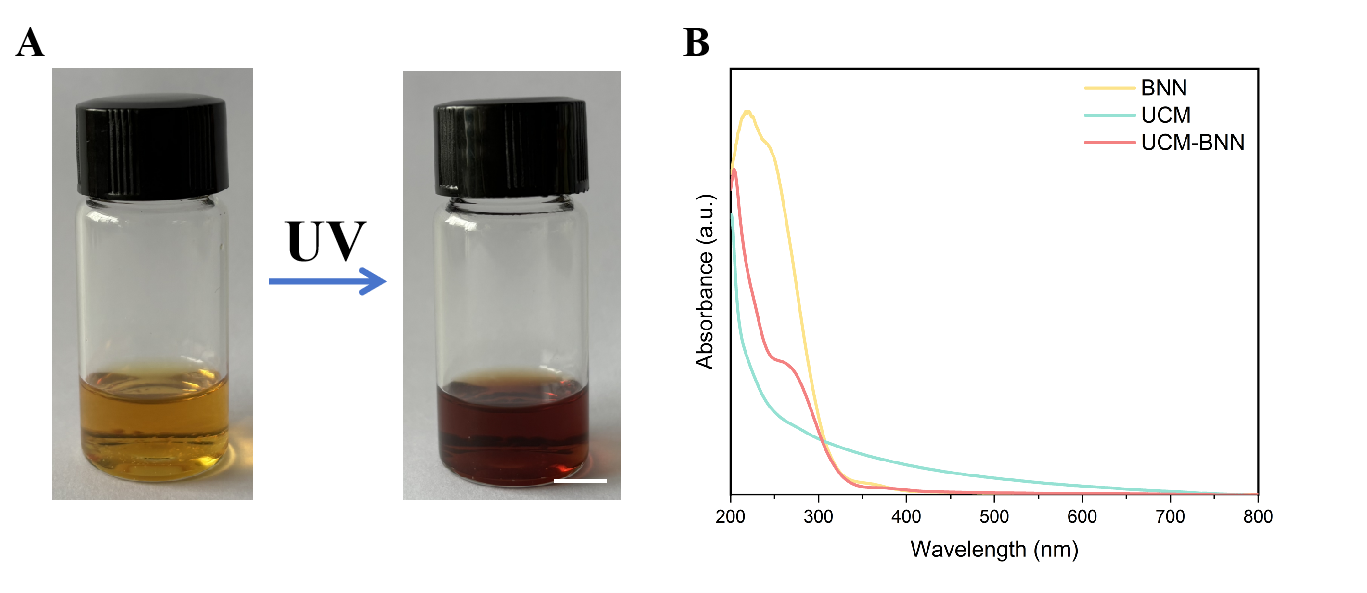


**Figure S4.** (A) Photodegradation process of BNN, where a color change from pale yellow to red-brown can be observed during the reaction; (B) UV-Vis absorption spectra of BNN, UCM, and UCM-BNN in absolute ethanol. (Scale bar: 1 cm).


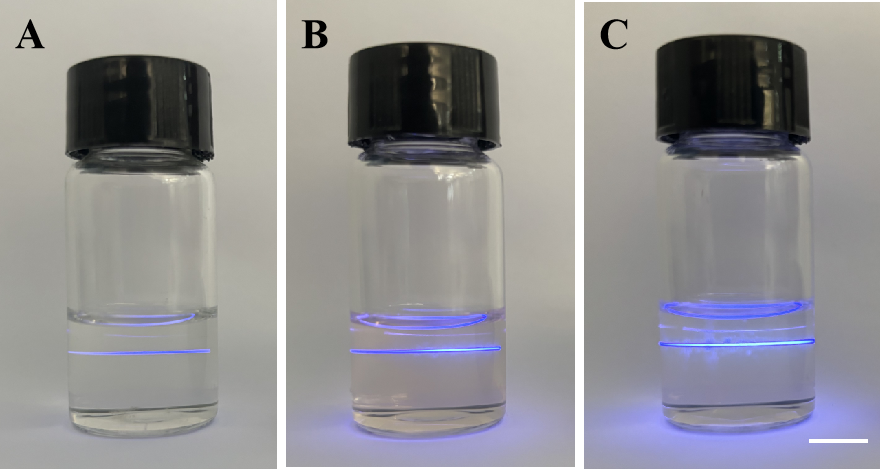


**Figure S5.** Upconversion luminescence images of (A) NaYF_4_:25%Yb, 0.5%Tm (core); (B) NaYF₄:25%Yb, 0.5%Tm@NaYF_4_:20%Nd, 10%Yb (core@shell) and (C) NaYF_4_:25%Yb, 0.5%Tm@NaYF_4_:20%Nd, 10%Yb@NaYF_4_ (core@shell@shell) under 980 nm NIR excitation. (Scale bar: 1 cm).


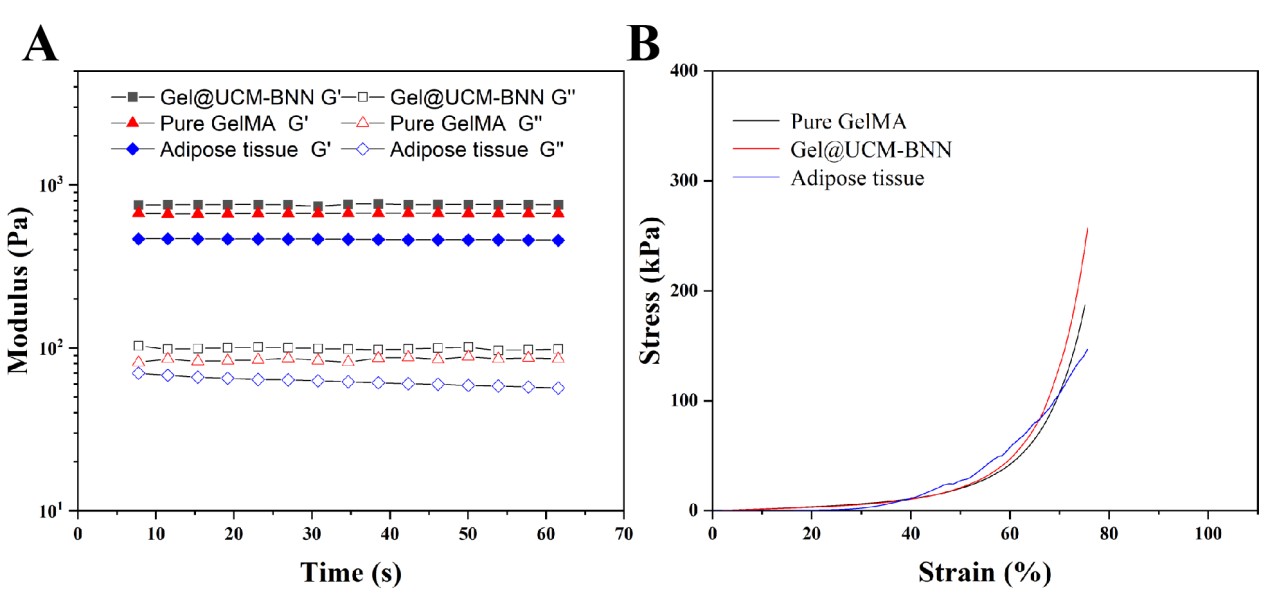


**Figure S6.** Rheological test results (A) and stress-strain curves (B) of pure GelMA, Gel@UCM-BNN hydrogel and adipose tissue.


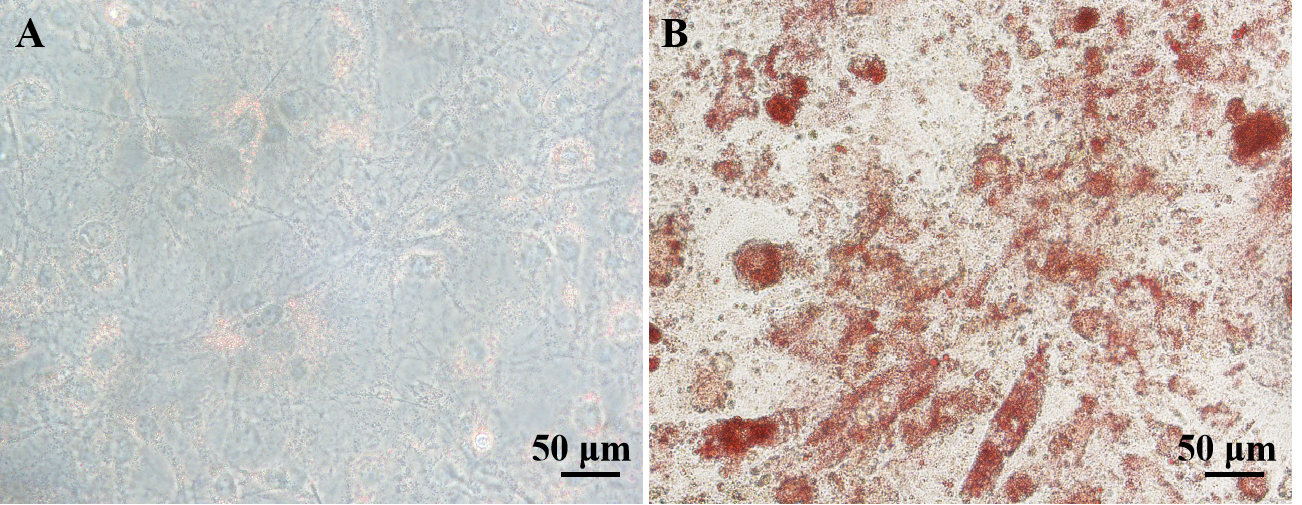


**Figure S7.** Microscopic images of 3T3-L1 cells (A) and 3T3-L1 cells induced to differentiate into adipocytes (B).


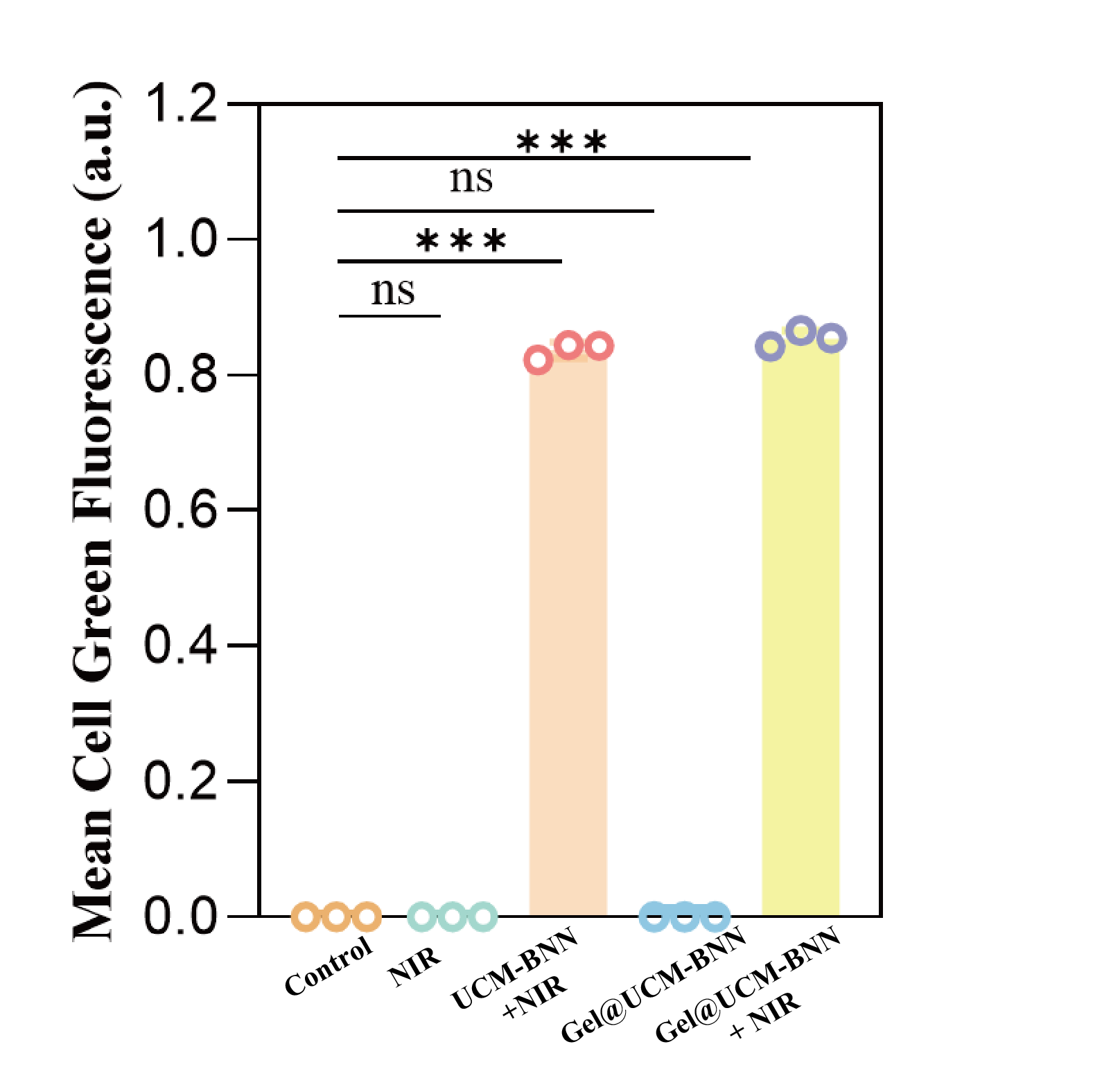


**Figure S8.** Quantitative analysis results of intracelluar NO release in adipocytes. Data are presented as mean ± standard deviation (n ≥ 3). (****p* < 0.001, ns means not significant).


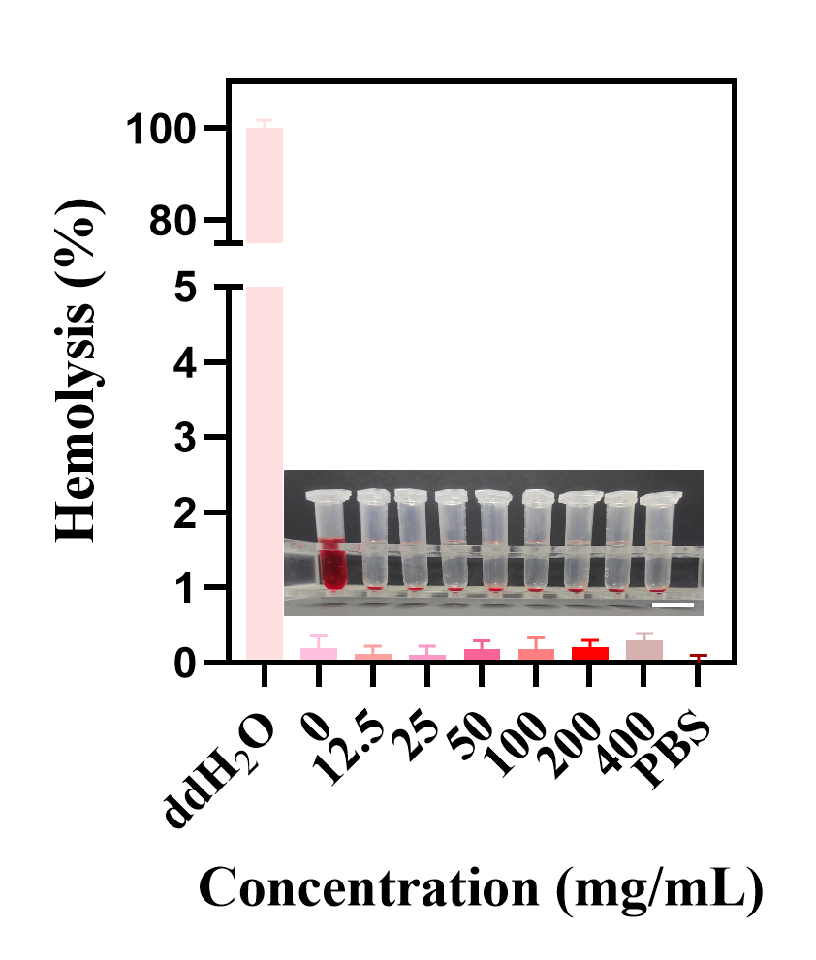


**Figure S9.** Hemolysis ratio of Gel@UCM-BNN hydrogel at different concentrations. Data are presented as mean ± standard deviation (n ≥ 3). (Scale bar = 1 cm).


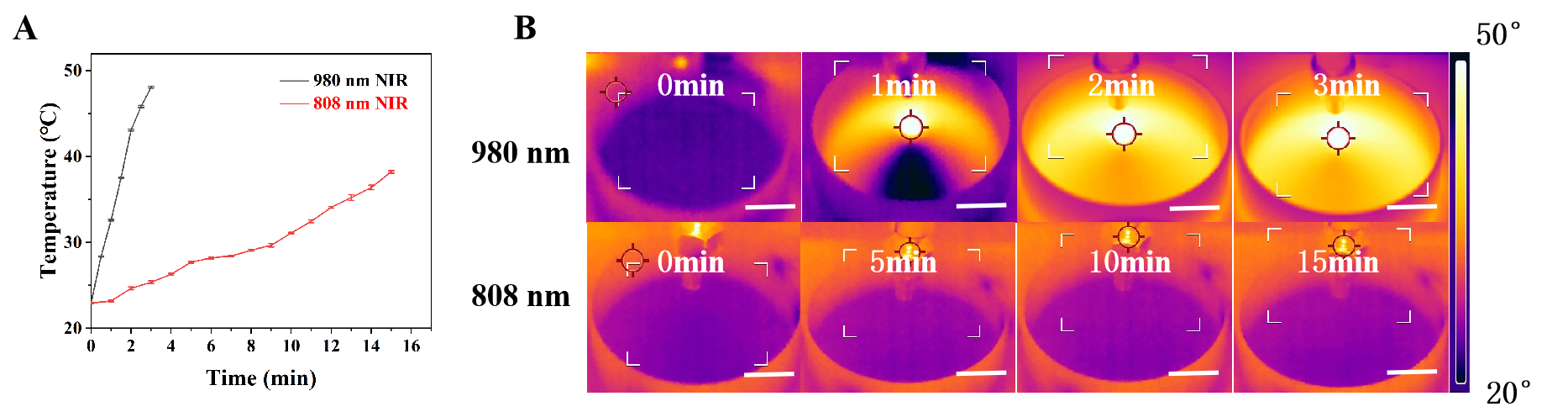


**Figure S10.** Heating curves (A) and thermal imaging photographs (B) of Ultrapure Water after irradiation with 980 nm and 808 nm lasers. Data are presented as mean ± standard deviation (n ≥ 3). (Scale bar: 1 cm).


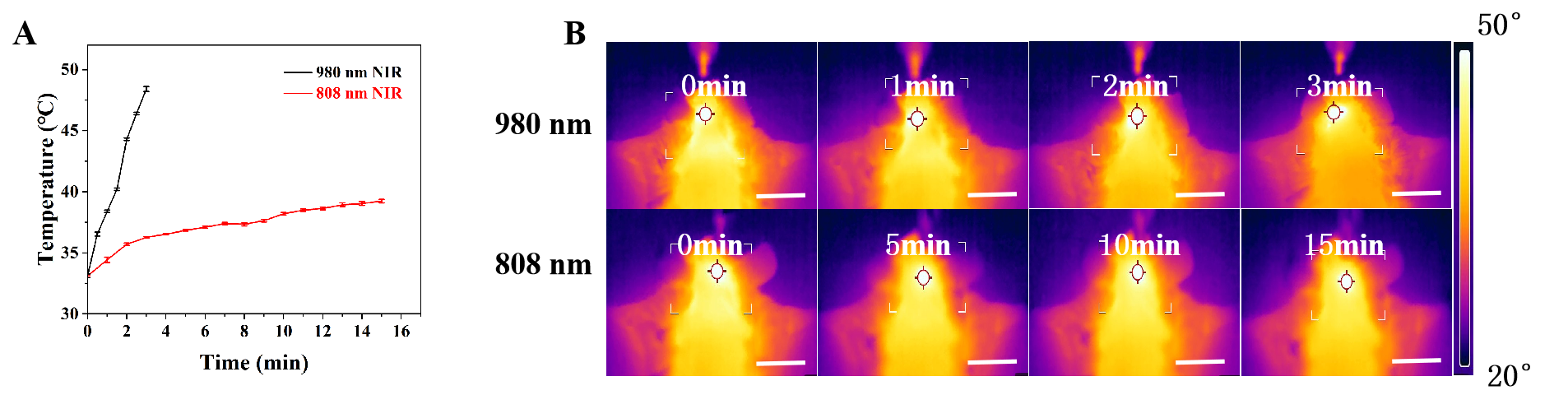


**Figure S11.** Heating curves (A) and thermal imaging photographs (B) of the skin on the top of mice's heads after irradiation with 980 nm and 808 nm lasers. Data are presented as mean ± standard deviation (n ≥ 3). (Scale bar: 1 cm).


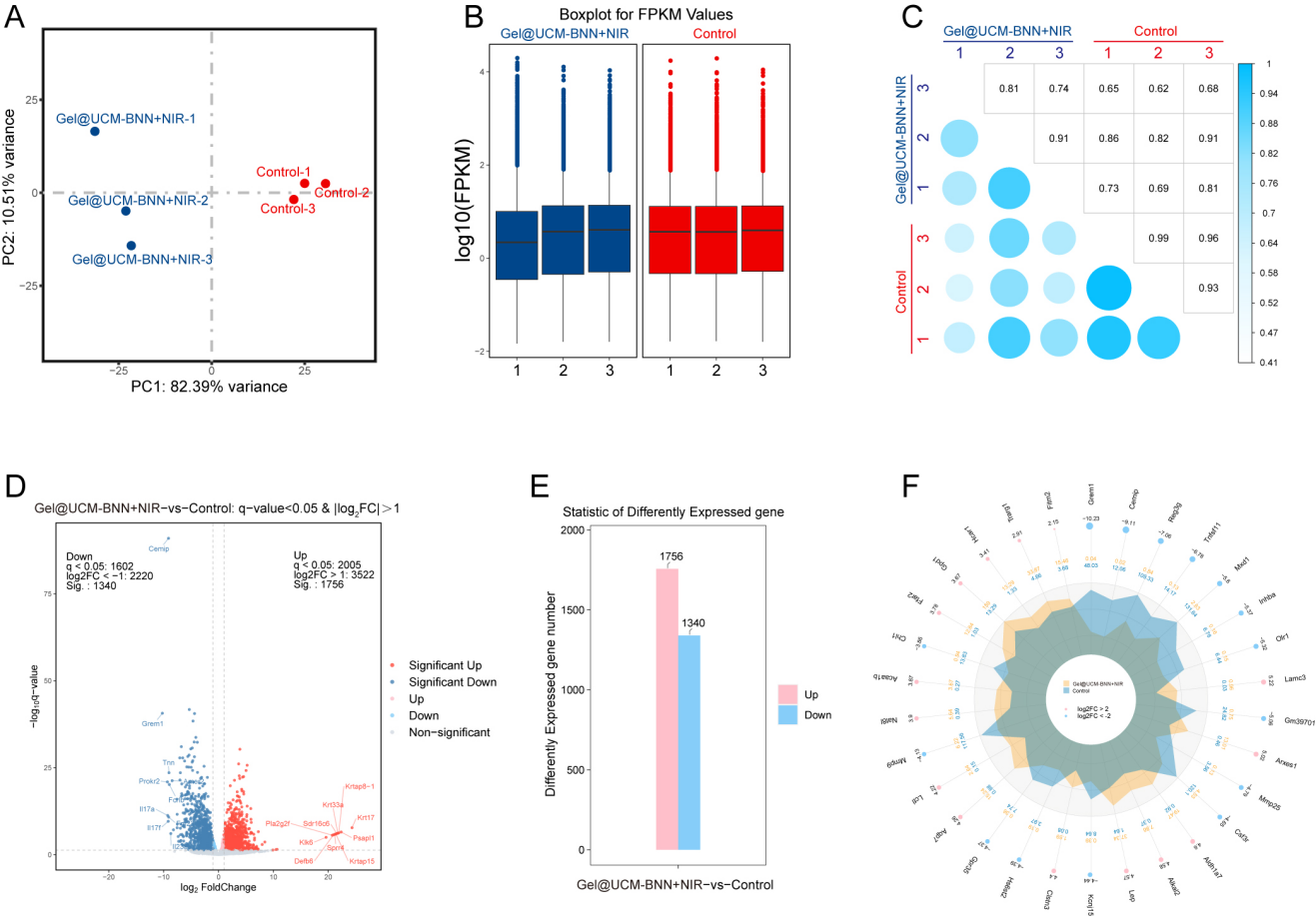


**Figure S12.** Quality control of RNAseq. (A) Principal components analysis suggests differences in sample gene distribution. (B) Box plot indicating RNA expression of each sample. (C) Correlation analysis suggests the similarity of gene expression trends among different samples. (D) Volcano plot displays differentially expressed genes between the Gel@UCM-BNN + NIR group and the control group. (E) Bar chart displaying the number of upregulated and downregulated genes between the Gel@UCM-BNN + NIR group and the control group. (F) Top 15 differential expressed genes between the Gel@UCM-BNN + NIR group and the control group.
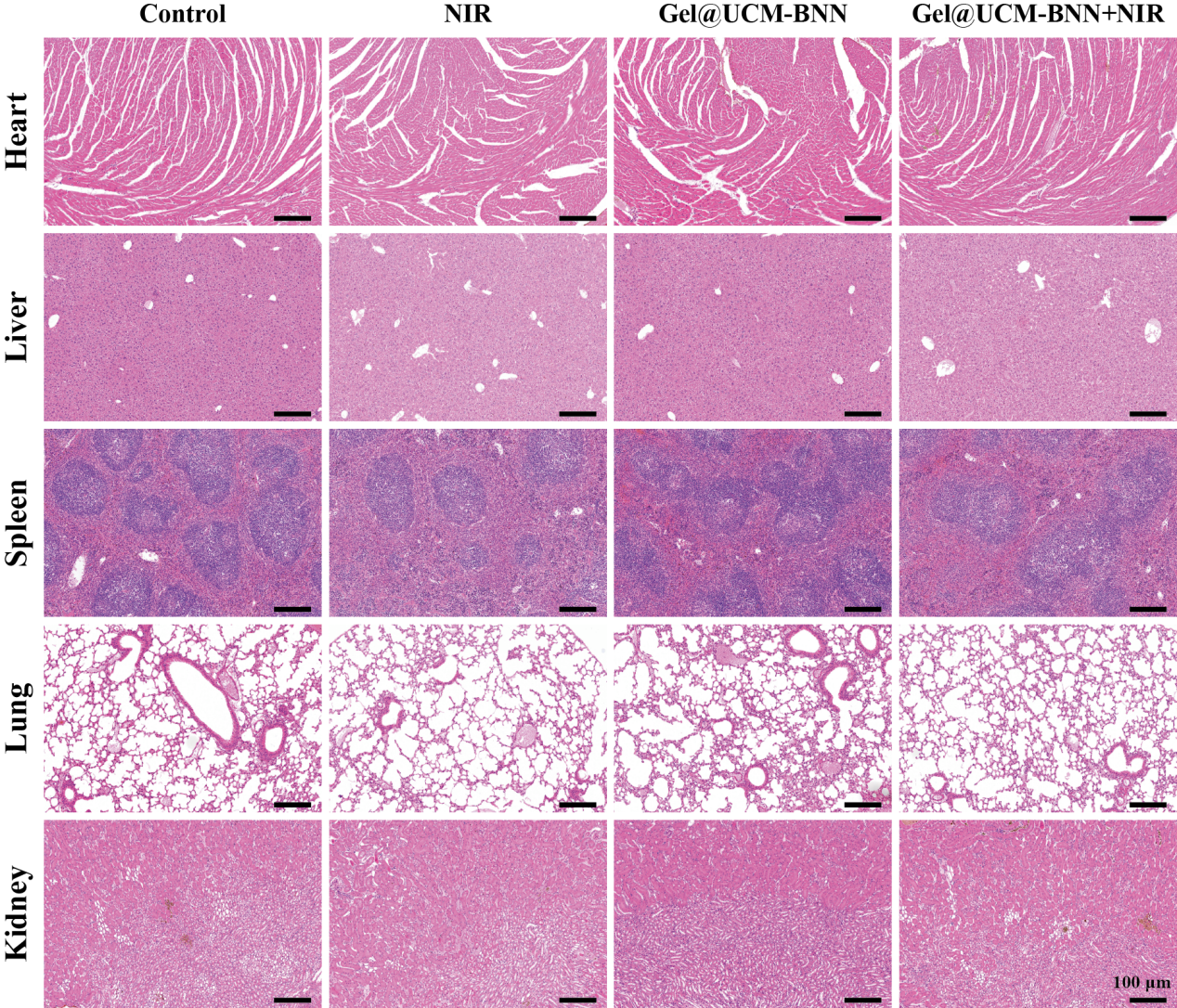


**Figure S13.** H&E staining images of major organs in different groups after treatment.


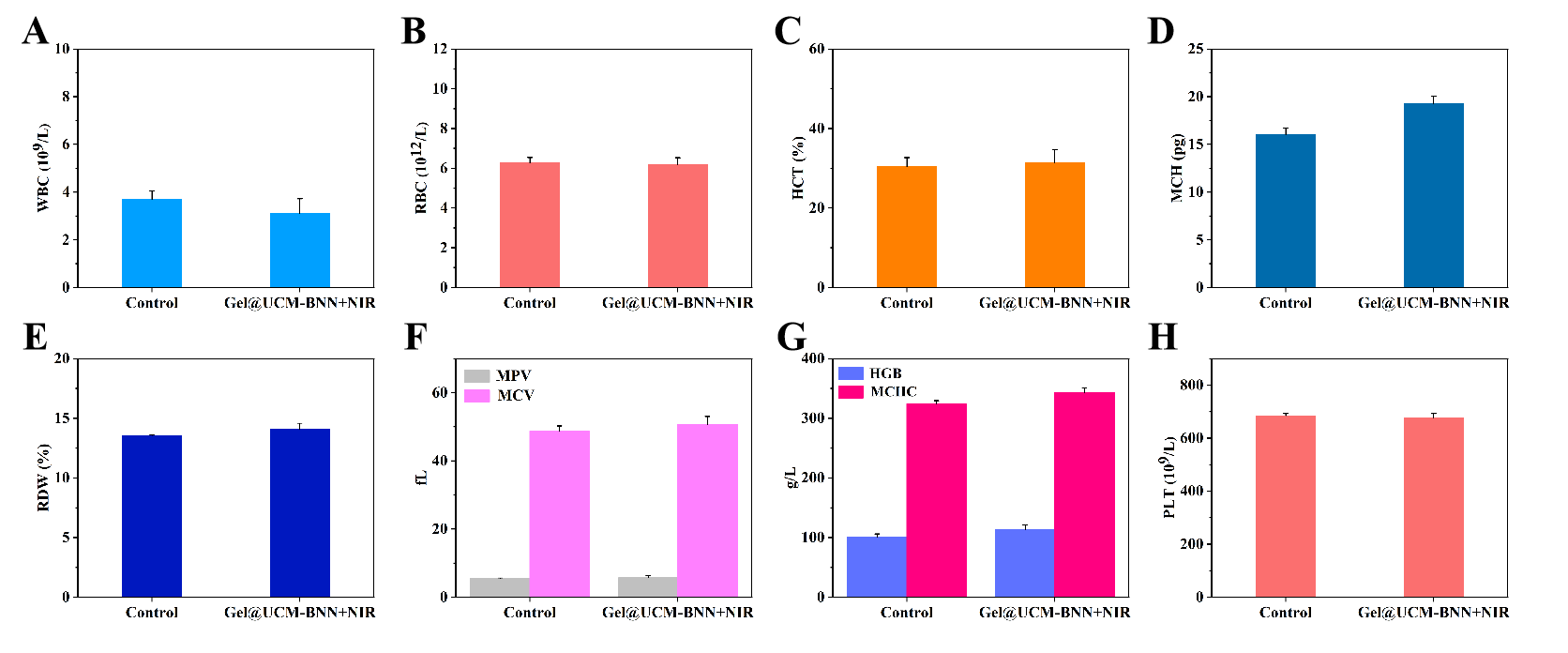


**Figure S14.** Blood cell analysis results (A-H) of mice in the control group and Gel@UCM-BNN + NIR group after treatment. Data are presented as mean ± standard deviation. (n ≥ 3).

**
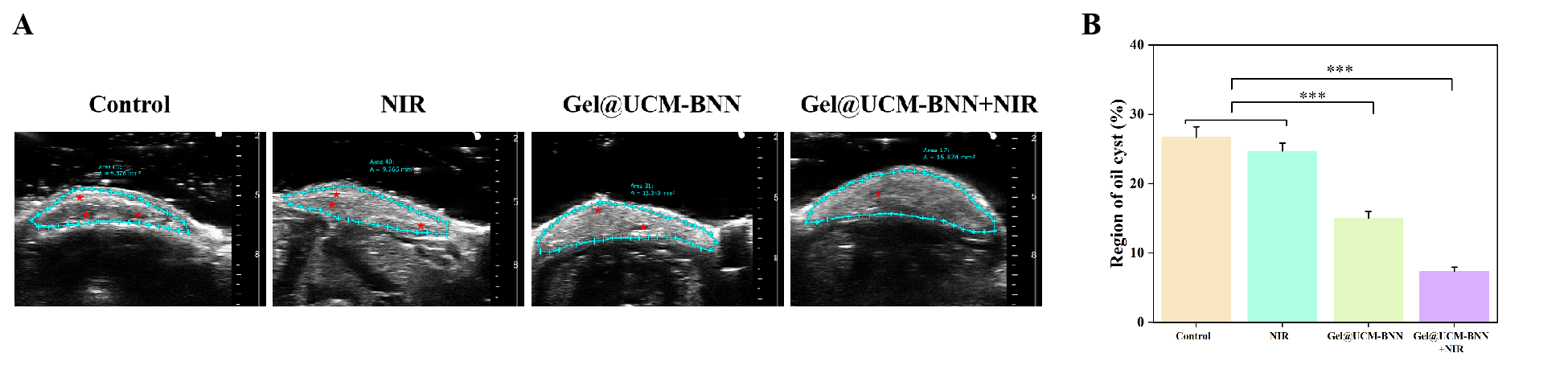
**

**Figure S15.** **Evaluation of fat grafts 12 weeks post-transplantation.** (A) Ultrasound examination results showing hypoechoic regions (oil cysts, marked with asterisks). (B) Quantification of oil cyst areas and their proportions. Data are presented as mean ± standard deviation. (****p* < 0.001, n ≥ 3).
